# Systematic use of synthetic 5′-UTR RNA structures to tune protein translation improves yield and quality of complex proteins in mammalian cell factories

**DOI:** 10.1093/nar/gkaa847

**Published:** 2020-10-13

**Authors:** Peter Eisenhut, Aman Mebrahtu, Mona Moradi Barzadd, Niklas Thalén, Gerald Klanert, Marcus Weinguny, Anna Sandegren, Chao Su, Diane Hatton, Nicole Borth, Johan Rockberg

**Affiliations:** ACIB Austrian Centre of Industrial Biotechnology, Krenngasse 37, 8010 Graz, Austria; BOKU University of Natural Resources and Life Sciences, Department of Biotechnology, Vienna 1190, Austria; KTH Royal Institute of Technology, Department of Protein Science, 10691 Stockholm, Sweden; KTH Royal Institute of Technology, Department of Protein Science, 10691 Stockholm, Sweden; KTH Royal Institute of Technology, Department of Protein Science, 10691 Stockholm, Sweden; ACIB Austrian Centre of Industrial Biotechnology, Krenngasse 37, 8010 Graz, Austria; ACIB Austrian Centre of Industrial Biotechnology, Krenngasse 37, 8010 Graz, Austria; BOKU University of Natural Resources and Life Sciences, Department of Biotechnology, Vienna 1190, Austria; Affibody Medical AB, Scheeles väg 2, SE-171 65 Solna, Sweden; SOBI AB, Tomtebodavägen 23A, Stockholm, Sweden; AstraZeneca, Biopharmaceutical Development, Milstein Building, Granta Park, Cambridge CB21 6GH, UK; ACIB Austrian Centre of Industrial Biotechnology, Krenngasse 37, 8010 Graz, Austria; BOKU University of Natural Resources and Life Sciences, Department of Biotechnology, Vienna 1190, Austria; KTH Royal Institute of Technology, Department of Protein Science, 10691 Stockholm, Sweden

## Abstract

Predictably regulating protein expression levels to improve recombinant protein production has become an important tool, but is still rarely applied to engineer mammalian cells. We therefore sought to set-up an easy-to-implement toolbox to facilitate fast and reliable regulation of protein expression in mammalian cells by introducing defined RNA hairpins, termed ‘regulation elements (RgE)’, in the 5′-untranslated region (UTR) to impact translation efficiency. RgEs varying in thermodynamic stability, GC-content and position were added to the 5′-UTR of a fluorescent reporter gene. Predictable translation dosage over two orders of magnitude in mammalian cell lines of hamster and human origin was confirmed by flow cytometry. Tuning heavy chain expression of an IgG with the RgEs to various levels eventually resulted in up to 3.5-fold increased titers and fewer IgG aggregates and fragments in CHO cells. Co-expression of a therapeutic Arylsulfatase-A with RgE-tuned levels of the required helper factor SUMF1 demonstrated that the maximum specific sulfatase activity was already attained at lower SUMF1 expression levels, while specific production rates steadily decreased with increasing helper expression. In summary, we show that defined 5′-UTR RNA-structures represent a valid tool to systematically tune protein expression levels in mammalian cells and eventually help to optimize recombinant protein expression.

## INTRODUCTION

In a living cell, gene expression levels and the resulting expression patterns are stringently controlled at many stages during the process of protein production. Unsurprisingly even subtle changes in protein expression levels may have profound impact on the cellular phenotype, i.e. during embryonical development (1) or progression of diseases (2,3). Hence, trying to investigate gene function(s) by artificially manipulating its expression level, i.e. by a knock-out or transgenic overexpression rarely represents physiologically relevant conditions. Consequently, studying and manipulating cellular phenotypes requires additional and more sophisticated precision tools to predictably tune expression levels.

The controlled manipulation of gene expression is also an important tool in biosynthetic engineering approaches to optimize the manufacture of desired products, such as biopharmaceuticals, in cellular organisms. Today, mammalian cells, such as Chinese hamster ovary (CHO) cells, are the predominant host cell lines used for the production of therapeutic proteins (4). While these cells have proven to have a cellular machinery well fit for production of monoclonal antibodies, i.e. IgGs, and several other human proteins for therapeutic usage, recent interests in more difficult-to-express human and artificial protein-fusions, have highlighted product yield and quality bottlenecks in CHO (5–7). Studies using transient expression in CHO cells have shown that product yield can be enhanced by regulating the expression of protein subunits and helper proteins (8,9). To further enhance the performance of mammalian cell lines or the specifically defined modification patterns of the protein product, it will be important to have tools that allow precise control of gene expression levels of product and helper genes or to introduce new genetic modules and complex genetic networks with balanced expression patterns (10,11).

Gene expression levels are regulated on many layers. Thus multiple interference points to intentionally tune expression levels exist (12). Tools to control the transcriptional activity by promoter engineering have been described for both prokaryotic and eukaryotic systems and classically function either by engineering of promoters themselves, e.g. adding transcription factor binding sites, or by directing synthetic transcription factors to the promoter of interest (13–16). Such synthetic promoters cover relatively broad dynamic ranges of expression levels and have been applied to improve recombinant protein titers in CHO cells (13,17). Yet, these strategies usually are dependent on the expression levels of endogenous transcription factors and, once stably integrated into the genome, also on the genomic environment. Hence, discrepancies in performance can arise if the toolbox is applied to different host cell types (18). In an alternative approach, methodologies to regulate mRNA levels at a post-transcriptional stage have been described for mammalian cells, e.g. by the incorporation of miRNA targets in the 3′-untranslated region (UTR) (19) or by riboswitches (20–22). Probably the most conserved step during protein production is translation. Consequently, a regulation at this stage may be preferable, as it will likely function similarly in various host cell types and different strategies have been investigated, i.e. altering the sequences of the translation initiation sites (TIS) (23) or introducing upstream open reading frames (uORF) (24). In fact, TIS variants have been very recently reported to improve quality of bispecific antibody production in CHO cells (25) while Ferreira *et al.* showed that their uORF strategy functioned comparably in different cell types (24).

RNA secondary structure elements in the 5′-UTR of a mRNA are known to impact translation rates, likely by impacting the speed that the DEAD-box helicase eukaryotic initiation factor 4A requires to unwind the structure (26–30). Babendure *et al.* showed that protein expression levels in mammalian cells are dependent on the thermodynamic stability, GC-content and relative position of RNA hairpins in the 5′-UTR (31) and similar findings were reported in yeast (32,33). Nonetheless, to our knowledge defined RNA hairpins have so far not been employed to intentionally tune protein expression levels for improved recombinant protein production in mammalian cell factories.

**Figure 1.**
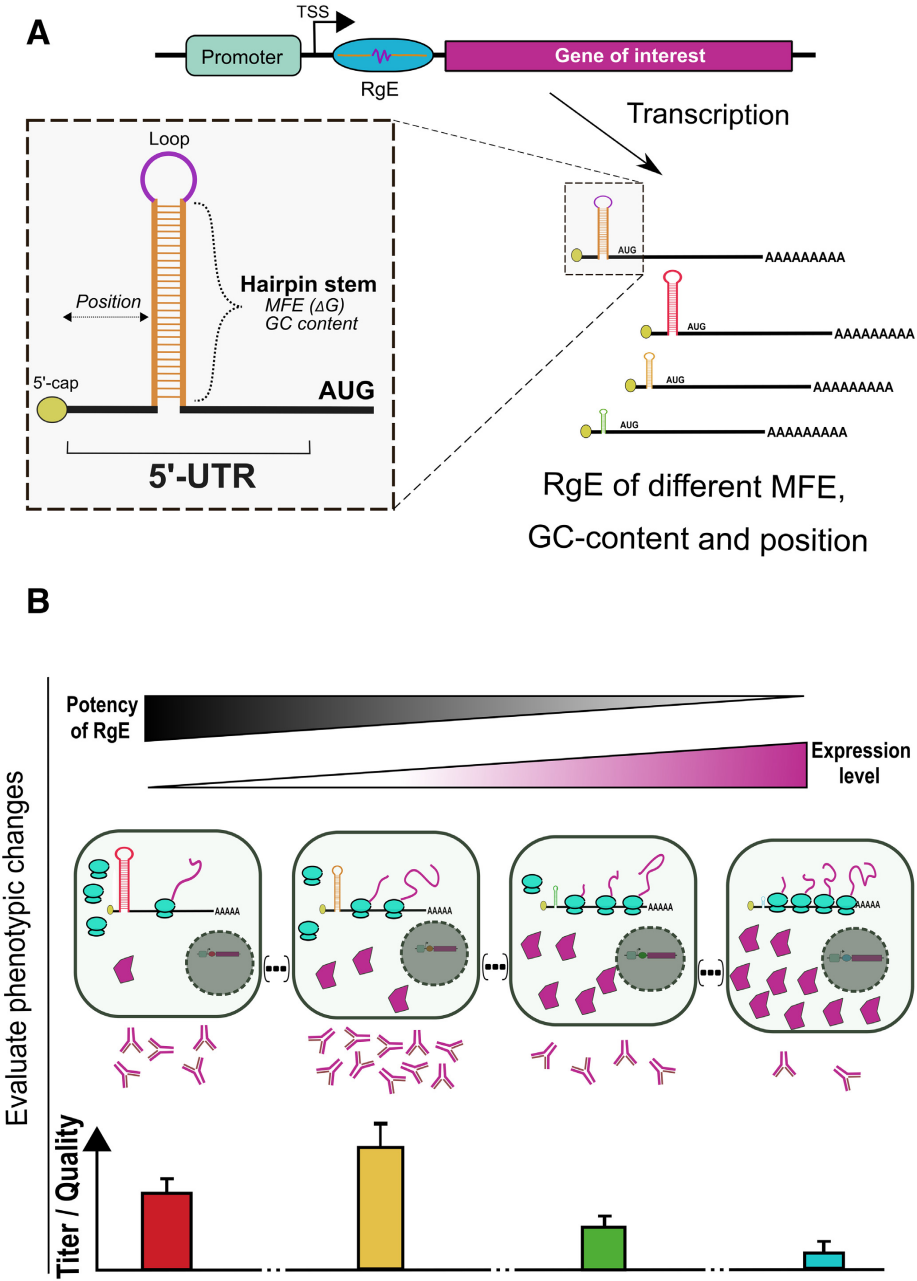
Schematic representations of regulation element (RgE) actions. (**A**) The RgE is cloned between the transcription start site (TSS) and the coding sequence of the gene to be regulated. When this gene is transcribed the RgE will fold and form an RNA secondary structure. The efficiency of the RgE can be influenced by changing the minimum free energy (MFE), the GC-content of the RNA stem and the relative position to the 5′-cap of the mRNA. (**B**) The underlying hypothesis is that using such RgE to tune expression levels of either the recombinantly introduced product itself or a helper factor can increase secretion of the desired product by mammalian cell factories by improving assembly or processing.

In this study we therefore sought to construct a panel of defined RNA hairpins, which we termed ‘Regulation element(s)’ or short ‘RgE(s)’, and characterize them for their ability to tune protein expression in two of the most commonly used mammalian expression hosts (principle shown in Figure 1), namely CHO-K1 cells and human embryonic kidney (HEK) 293 cells. To show the applicability of RgEs in two specific cell engineering problems, we chose to employ a set of these RgEs to (i) tune expression levels of the subunits of a multimeric protein and (ii) of a required helper protein in the second case. For the optimal expression of recombinant IgG in CHO cells, it is well-known that often heavy chain (HC) levels should be reduced to optimize IgG assembly (34–36). Thus, this case served as a good proof-of-principle for our RgEs. The second case addresses the expression of the recombinant Arylsulfatase A (ASA) which requires a unique post-translational modification of a cysteine to a C_α_-formylglycine in its active site to be catalytically active. This modification is catalyzed by the enzyme encoded by the sulfatase modifying factor 1 (SUMF1) gene (37,38) which is one of the limiting factors for active ASA production. Therefore, co-expression of SUMF1 is required to enhance the activity of recombinantly produced ASA. The here described findings highlight the importance of regulating protein expression levels to control cellular performances and product traits. Eventually, this study should facilitate the use of the RNA hairpin toolbox to intentionally and predictably control protein expression levels in mammalian cells to enhance recombinant protein production or study gene dosage functions.

## MATERIALS AND METHODS

### Regulation element (RgE) design

The sequences of the RgEs and the kissing hairpins (‘KH’) were designed manually, based on structure and minimum free energy (MFE, ΔG) predictions by the RNAfold web server (39) and GC-content calculations of the hairpin stem region. All sequences are provided in Supplementary Table S1. Restriction enzyme-based cloning was used to clone the RgEs (or KHs) in the 5′-UTR (described in more detail below). The transcription start site (TSS) from a CMV promoter was defined as reported by Isomura *etal* (74). By default, an enzyme that cleaves at position +15 bp, relative to the TSS, was used. To place RNA structures at positions other than +15 bp, either sequence stretches from the upstream 5′-UTR up to position +7 or +3 were included in the hairpin design, or a spacer sequence of 5′- CAA -3′ repeats, that should not form any secondary structures (31), was added to place it at position +33. All RgEs were ordered as single-stranded DNA oligos (IDT, Inc., USA or Eurofins Scientific, Luxemburg) with the correct overhangs required for cloning and then annealed as described in Bauer *et al.* (40). Similarly, short hairpin RNA regulation elements (RgE), previously evaluated siRNAs sequences for Rad21, Chd4 (41), Dnmt1 and Dnmt3a (42) were used as RgEs (see Supplementary Figure S8b for sequences). Noteworthy, the ordered oligos contained only the required overhang sequences and not the full restriction enzyme recognition sequence. Also, these overhang sequences were not considered in MFE predictions by RNAfold, only the actual hairpin stem and loop sequences where used.

### Construction of expression vectors

To construct the plasmid pTagRFP-TagBFP, the TagRFP gene with CMV promoter and enhancer was amplified from the plasmid pTagRFP-C (# FP141, Evrogen, Russia) with the TagRFP_fwd and -_rev primer. The amplicon was then digested with PciI (New England Biolabs, USA) and ligated into the PciI restricted plasmid pTagBFP-C (# FP171, Evrogen). Next, the annealed RgE oligos were ligated into AfeI (New England Biolabs) and AgeI (New England Biolabs) digested pTagRFP-TagBFP, yielding the final expression vectors. To construct the trastuzumab expression vectors, the selected RgEs including the CMV promoter were amplified with the primers traz_fwd and -_rev from the beforehand generated expression vectors and cloned into NotI (New England Biolabs) and PmeI (New England Biolabs) restriction sites in the 5′-UTR of the HC gene in the vector (pKTH17_trastuzumab, harboring the genes for heavy and light chain). To generate SUMF1 expression constructs a SacI restriction site on the SUMF1 plasmid, originally established by solid phase cloning (43,44), was first removed to have a single SacI site in the 5′-UTR to allow for directional cloning of RgEs. Therefore, the vector pKTH16_sumf1 was digested with HindIII-HF (New England Biolabs) and XhoI (New England Biolabs) and ligated with beforehand annealed oligos SUMF1_SacIX_fwd and SUMF1_SacIX_rev, that carry a point mutation in the SacI site. Next, the annealed RgE oligos were ligated into a SacI (New England Biolabs) and NotI (New England Biolabs) digested plasmid yielding the SUMF1 expression vectors. All DNA sequences used for cloning are available in Supplementary Table S2.

### Cell culture

CHO-K1 cells (ECACC 85051005) adapted to grow in suspension were routinely cultivated in CD-CHO medium (Thermo Fisher Scientific, USA) supplemented with 8 mM L-glutamine (Thermo Fisher Scientific) and anti-clumping agent (1:500 diluted, Thermo Fisher Scientific) and incubated at 37°C, 5% CO_2_, humidified air and 120 rpm shaking. Cells were passaged every 3–4 days by dilution to a cell density of 2.0 × 10^5^ cells/ml in 25 ml fresh medium in a 125 ml shaker flask (Corning^®^, Sigma Aldrich, USA). HEK Freestyle™ 293-F cells (Thermo Fisher Scientific) were cultivated in Freestyle™ 293 expression medium (Thermo Fisher Scientific) without further supplements, incubated and passaged under the same conditions as CHO-K1 cells. ExpiCHO™ cells (Thermo Fisher Scientific) were routinely cultivated in ExpiCHO™ expression medium (Thermo Fisher Scientific), passaged according to the manufacturer's instructions and incubated at 37°C, 5% CO_2_, humidified air and 120 rpm.

### Transfection

Both, CHO-K1 and HEK293 cells were transfected with polyethylenimine (‘PEI’) MAX (Mw 40000) (Polysciences, Inc., USA). For the CHO-K1 transfections, a total of 2.5 * 10^6^ cells were centrifuged at 170 rcf for 5 min and resuspended in 2.5 ml of the respective medium without anti-clumping agent and transferred to one well of a 24 deep-well plate (CR1424cl, EnzyScreen BV, Netherlands). A total of 2.5 μg of plasmid DNA were diluted in 100 μl OptiPRO™ SFM (Thermo Fisher Scientific). A total of 15 μg PEI MAX (c = 1 mg/ml), ratio of plasmid:PEI = 1:6, were diluted in 100 μl OptiPRO™ SFM, mixed with the plasmid solution, incubated at room temperature for 15 min and then slowly added to cells. The cells were incubated at 37°C, 8% CO_2_, humidified air and 250 rpm shaking. The transfections of HEK cells were performed in the same way, but: 2.0 * 10^6^ cells were resuspended in 2 ml Freestyle™ 293 expression medium, 2.4 μg plasmid DNA and 9.6 μg PEI MAX (1:4 ratio) were used per transfection. The cells were incubated under the same conditions as the CHO-K1 cells. ExpiCHO™ cells were transfected as suggested by the manufacturer with a total of 20 μg trastuzumab plasmid DNA per transfection. For the ASA/SUMF1 co-expressions, again ExpiCHO™ cells were transfected as suggested by the manufacturer with 17 μg of the ASA expression constructs plus 20 μg of the SUMF1-regulation plasmids.

### Flow cytometry

The expression of the fluorescent proteins was evaluated by recording 1.5 * 10^5^ events in a Gallios™ flow cytometer (Beckman Coulter, USA). Cells were gated for viability based on forward and side scatter and a side scatter area to height gate to identify single cells. TagBFP (BFP) was excited by a violet 405 nm laser and detected with a 450/50 bandpass (BP) filter. TagRFP (RFP) was excited with an argon 488 nm laser and detected with a 620/30 BP filter. Analysis of results was done using the Kaluza flow cytometry analysis v2.1 software (Beckman Coulter). The ratio of red fluorescent protein (RFP) to blue fluorescent protein (BFP) was determined at the single cell level. Therefore, information for cells that were successfully transfected (gate on BFP-positive cells) was exported from Kaluza and the ratio of the fluorescent signal recorded for RFP to the fluorescent signal recorded for BFP was calculated for each individual cell. Next, the geometric mean was calculated from all individual ratios. Geometric mean values of the different RgEs were then compared to the geometric mean of the unregulated CMV control to determine the fold changes (FC) of RFP expression levels. The work flow and gating strategy is depicted in Supplementary Figure S1.

### Quantitative real-time PCR

To determine differences in RFP and BFP mRNA levels, 5 * 10^6^ cells of both CHO and HEK were harvested on day 2 after the transfection, centrifuged at 170 rcf for 8 min and resuspended in 300 μl RNAprotect^®^ cell reagent (Qiagen, Netherlands) and stored at −20°C until further isolation. The total RNA was purified using RNeasy^®^ plus mini kit (Qiagen) according to the manufacturer's instructions. Genomic DNA was removed by the provided gDNA eliminator columns according to the manufacturer's protocol. Concentration and purity (260/280 ratio) of the purified RNA samples was determined in a NanoDrop™ 2000 (Thermo Fisher Scientific) with the pre-installed program to determine RNA concentrations. cDNA was then generated from 800 ng isolated RNA (260/280 ratio of all samples ∼2) using the High-Capacity cDNA reverse transcription kit (Thermo Fisher Scientific) with RNase inhibitor (20 U/L) (Thermo Fisher Scientific) according to the manufacturer's protocol. The cDNA samples were then diluted 1:4 in nuclease-free water and stored at −20°C until used. Quantification of each cDNA template was performed in quadruplicates (or triplicates in case of the shRNA-RgE screening) on the CFX96™ real-time system (Bio-Rad Laboratories, USA). A total of 1 μl cDNA was mixed with 5 μl 2× iQ™ SYBR^®^ green supermix (Bio-Rad Laboratories), 0.5 μl 10 μM forward primer, 0.5 μl 10 μM reverse primer and 3.5 μl nuclease-free water. All primers used to quantify relative gene expression are provided in Supplementary Table S3. Gapdh was used as a reference gene in CHO cells and the HPRT1 pre-designed quantitative polymerase chain reaction (qPCR) assay (Hs.PT.58v.45621572, IDT, Inc.) was used in HEK. Cycling conditions were 95°C 2 min, 40 cycles of 95°C 15 s, 60°C 20 s and 72°C 20 s, and a melting curve was recorded from 65 to 99°C 0.5°C/step at 2 s for each step to monitor specific amplifications. Reverse transcription controls (no reverse transcriptase added) and no template controls were included and showed no signs of contaminations. cT values were determined with the CFX Maestro™ Software (Bio-Rad Laboratories). Gene expression levels were relatively quantified with the 2^−ΔΔCT^ (45). The ratio of RFP to BFP mRNA was then calculated and fold changes were related to the unregulated CMV (‘CMV’) transfected control. Relative changes of the RNAi target mRNAs levels were determined in the same as the RFP and BFP mRNA expression levels.

HC mRNA levels were quantified in a similar fashion with few modifications. Briefly, <10^7^ ExpiCHO™ cells were harvested at the end of the expression batch, centrifuged at 170 rcf for 8 min and the cell pellet resuspended in 300 μl RNAprotect^®^ cell reagent (Qiagen) and stored at −20°C until further isolation. RNA was isolated with the RNeasy^®^ mini kit (Qiagen) according to the manufacturer's instructions and gDNA was removed by on-column digestion with DNase I as suggested by the kit's manufacturer. cDNA was generated as described before. To quantify HC gene expression levels a 2× SensiFAST SYBR^®^ Hi-ROX mix (Bioline, UK) and a Rotor-Gene Q (Qiagen) machine was used. qPCR mix and cycling conditions were as described before. Gapdh was used as reference gene. cT values were determined in the Rotor-Gene Q software (Qiagen) and 2^−ΔΔCT^ method (45) used to relatively quantify expression differences.

### IgG quantification

IgG concentrations in the supernatant were determined by bio-layer interferometry measurements in an Octet^®^ RED96e system (Fortébio Biologics by Molecular Devices, USA) with Dip and Read™ Protein A and Dip and Read™ Protein L biosensors (Fortébio Biologics by Molecular Devices) according to the manufacturer's instructions. The supernatant samples from day 4 and 5 were diluted 1:2 with phosphate-buffered saline +0.1% Tween and the samples from day 6 and 7 were diluted 1:4. A standard curve was prepared from an IgG with the respective concentrations of 1000 to 1 μg/ml.

### IgG purification

Antibodies were purified by utilizing Protein A magnetic beads (Thermo Fisher Scientific) and Magnatrix 8000+ pipetting robot (NorDiag AS, Norway). A total of 50 μl Protein A coated magnetic beads were washed in TBS-Tween 20 (1%) (TBS-T) buffer followed by incubation with 200 μl supernatant for 1 h. Beads were then collected and washed again in TBS-T. IgGs were eluted by incubation for 10 min in 100 μl 0.1 M glycine (pH 2.5) buffer and neutralized by addition of 10 μl 1 M Tris–HCl (pH 8.5). For size exclusion chromatography analysis, the expressed antibodies were purified by Protein A facilitated purification on an ÄktaSTART system (GE Healthcare, USA) using mAbSelect SuRe columns (GE Healthcare). A 20 mM sodium phosphate, 0.15 M sodium chloride (pH 7.3) buffer was used as binding and wash buffer, 0.1 M glycine (pH 2.5) as elution buffer and 1M Tris–HCl (pH 8.5) as neutralization buffer.

### Size exclusion chromatography

Purified IgGs were buffer exchanged into phosphate-buffered saline (PBS) buffer using Amicon 3 kDa MWCO (Merck Millipore, USA) centrifuge filtration according to manufacturer's instructions prior to size exclusion chromatography (SEC) analysis. In total, 20 μg IgG in 70 μl were injected onto a Superdex Increase 200 10/30 GL gel filtration column (GE Healthcare) coupled to an Agilent 1200 series HPLC system (Agilent Technologies, USA). SEC runs were performed at a 0.5 ml/min flow rate with PBS as a running buffer. Eluted protein fragments were detected by an online 280 nm absorption measurement. Data analysis and peak integrations were performed using GraphPad prism 8.0 (GraphPad Software, USA).

### Purification and activity measurement of Arylsulfatase A

ASA was purified from supernatants harvested from day 8 post transfection. The purification was carried out on a ÄKTAxpress (GE Healthcare) with an 1 ml CaptureSelect™ C-tag pre-packed column (Thermo Fisher Scientific) and 3 × 5 ml HiTrap desalting columns (GE Healthcare). All supernatants (30 ml) were filtered through a 0.45 μm filter before loading. First, buffer A (25 mM Tris, 150 mM sodium chloride, pH 7.0) was used for equilibration, sample application and washing and a 50 mM HAc (acetic acid) buffer (pH 2.5) was used to isocratically elute ASA. Secondly, eluted ASA was directly desalted through the HiTrap desalting columns and eluted in buffer A. Elution fractions containing protein were pooled (measured by 280 nm absorption) and concentrated using Amicon Ultra-4 30 kDa MWCO (Merck Millipore) by centrifugation at 4000 rcf for 30 min. The concentration of the purified ASA was calculated by measuring the absorbance at 280 nm in a NanoDrop™ 2000 (Thermo Fisher Scientific) and using the extinction coefficient of 39350 (1/M cm) and a molecular weight of 52.677 kDa of ASA.

ASA activity was determined according the protocol described by Lee-Vaupel and Conzelman (46). Briefly, ASA converts the artificial chromogenic substrate 4-nitrocatecholsulfate (PNCS) to 4-nitrocatechol (PNC). This conversion was detected by determination of the absorbance at 515 nm. The amount of PNC generated was obtained by quantification with a PNC standard curve, and thus allows to determine the (specific) ASA activity in the respective samples.

### SDS-PAGE and western blot analysis

A total of 4 μg of the purified IgG samples were mixed with 3× loading buffer (0.1 M Tris–HCl, 45% glycerol, 0.03% bromophenol blue, 0.3% SDS) for non-reducing conditions and for the reducing analysis mixed with 3× loading buffer containing 0.15 M Tris 2-carboxyethyl-phosphine hydrochloride and incubation at 95°C for 10 min. The samples were run on a 4–20% Criterion™ TGX Stain-Free™ protein gel (Bio-Rad Laboratories) according to the company's protocol. The bands were visualized by staining the gel in GelCode™ Blue Safe protein stain (Thermo Fisher Scientific) for 1 h at room temperature and gentle shaking. Quantification of band ratios was performed in ImageJ Fiji. Western blot analysis was performed as recently described (47). Intracellular proteins were isolated from 5*10^6^ cells with Pierce^®^ RIPA buffer (Thermo Fisher Scientific) according to the manufacturer's instructions. All antibodies used are provided in Supplementary Table S4. The blot was visualized using the Odyssey^®^ imaging system (Li-Cor Biosciences, USA).

Screening SUMF1 expression levels via western blot was done in principle as described above. Cells (∼3*10^6^ cells) and supernatant were harvested on day 4 post-transfection. Supernatants were transferred to new tubes and cell pellets were lysed with M-PER™ mammalian protein extraction reagent (Thermo Fisher Scientific) according to the manufacturer's instructions. TBS-T (10 mM Tris, 150 mM NaCl, 0.05% Tween20, pH 7.5) and TBS-T + 5% milk powder were used as washing and blocking solution. Antibodies used are provided in Supplementary Table S4. SUMF1 bands were detected with Immobilon western HRP substrate (Merck Millipore) according to the manufacturer's instructions and imaged in a ChemiDoc XRS+ system (Bio-Rad Laboratories). SUMF1 levels were quantified by determination of the band intensities in ImageJ Fiji. The obtained values were normalized against the band intensity of the 55 kDa band of the Thermo Scientific™ PageRuler™ Plus prestained protein ladder, and then normalized to the viable cell density (from day 4 post-transfection, see Supplementary Figure S12) in case of the cell lysates or the cumulative cell days (from day 0–4, calculated as recently described (41)) in case of the supernatant, as the secreted factors accumulate over time. Lastly, these values were combined and relative SUMF1 levels were calculated in comparison to the unregulated CMV control.

### Statistical analysis

Evaluation of statistical differences was done in R version 3.5.2 (48). When applicable, the samples were first analyzed for homogeneity of variances with a Levene′s test. A one-way ANOVA was used to determine statistical differences and, where applicable, a Dunnett′s test was used as post-hoc test (*α* = 0.05) to determine statistical differences to the unregulated CMV samples. A minimum of three replicates were used for the statistical analysis. The coefficient of determination (*R*^2^) was calculated by linear regression analysis. The R package of ggplot2 was used to visualize data (49). Error bars in the graphs show standard error of mean (SEM) unless noticed differentially in the figure legends. ‘n.s.’ indicate non-significant differences, ‘#’ indicates a *P*-value < 0.1 and significant differences are indicated by ‘*’ *P*-value < 0.05, ‘**’ *P*-value < 0.01 and ‘***’ *P*-value < 0,001.

## RESULTS

### 5′-UTR RNA secondary structures predictably tune protein expression levels

**Figure 2.**
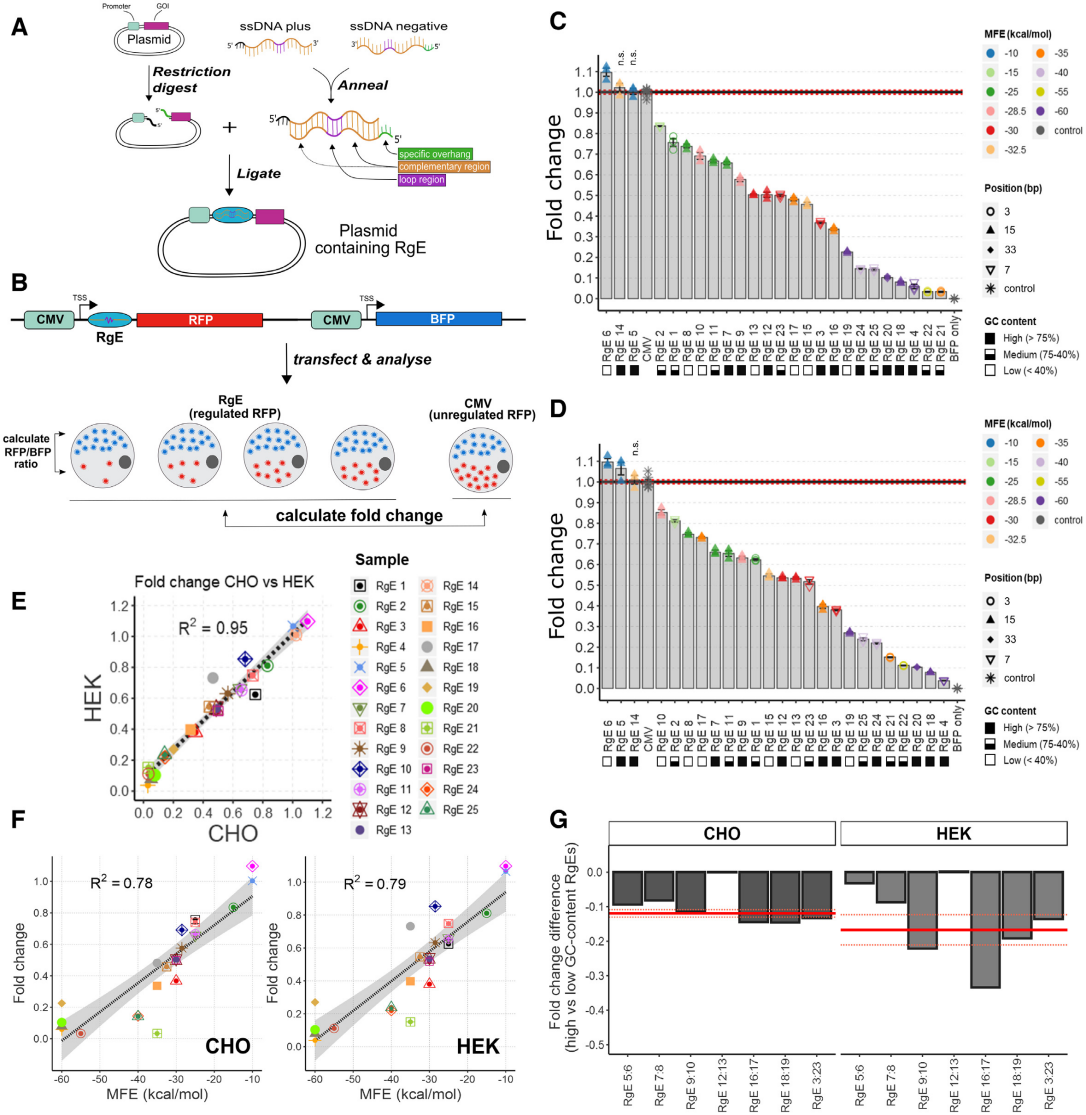
Proof-of-principle and evaluation of RgEs in mammalian cell lines. (**A**) Cloning procedure of RgE insertion into 5′-UTRs. GOI = gene of interest. (**B**) Schematic representation of experimental proof-of-principle. (**C** and**D**) Calculated fold change (FC) of RFP/BFP expression ratio mediated by RgEs screened in CHO (C) and HEK293 (D) cells in reference to the unregulated CMV sample. Bars show average, colored points show individual values of samples (*n* = 3 independent samples each; CMV: *n* = 6) and error bars show SEM. Color, shape and GC-content indicated on the right side. Elements are ordered according to their average fold change. The horizontal black line shows average, red dotted lines show SEM of CMV samples. All samples, except the ones marked with ‘n.s.’, showed a significant difference with *P* < 0.001 in comparison to the CMV sample. (**E**) Average fold changes of CHO versus HEK cells. Gray area shows standard error. (**F**) Average fold changes (FC) of CHO (left) and HEK (right) cell plotted against the MFE of the respective elements. Gray area as in (E). (**G**) Differences in fold change observed between high and low GC content elements of similar MFE at the same relative position. Y-axis depicts the calculated FC difference. X-axis shows the RgE pairs that were compared. Red full line shows averages (RgE 12:13 not included). Light red dotted line depicts SEM.

One of the major aims of this study was to generate a panel of RNA secondary structure elements for the 5′-UTR termed ‘RgEs’, that allow for predictable tuning of protein expression levels in mammalian cells. Therefore, 25 distinct RNA secondary structure elements differing in (i) the thermodynamic stability (MFE, ΔG), (ii) the GC-content of the hairpin stem and (iii) the position relative to the 5′-cap of the mRNA were designed (Figure 1A), as suggested by Babendure *et al.* (31). To evaluate and characterize the efficacy of these RgEs, they were cloned into the 5′-UTR of a RFP gene. A BFP gene was integrated on the same plasmid for normalization of transfection efficiency and expression levels (Figure 2A and B). These constructs were then transiently introduced into CHO-K1 and Freestyle™ HEK 293F cells. The expression levels of the two proteins were determined by flow cytometry of the transfected cells 2 days post-transfection and compared to a CMV control, in which RFP expression was not regulated (Supplementary Figures S1 and 2). We found that the designed RgEs are able to regulate protein expression levels as we observed a good coverage of the expression range from almost zero up to the initial promoter activity (CMV sample) (Figure 2C and D). A slight increase of the expression by 1.1-fold depending on the expression host could also be observed (RgE 6 (in both) and 5 (only in HEK)). The lowest levels of RFP were ∼ 0.02-fold in CHO (RgE 21) or 0.05-fold in HEK (RgE 4), respectively. Sequence confirmation revealed that RgE 14 had only half of the RgE sequence present (potentially due to faulty plasmid reproduction in the *Escherichia coli*), thus no functioning hairpin structure could form after transcription and no change in RFP expression levels was observed. RgE 14 was thus removed from further analyses. Comparison of the expression hosts demonstrates that the generated elements function in a comparable manner across cell lines from different mammalian organisms (*R*^2^ = 0.95) (Figure 2E). In general, we found that the synthetic RNA hairpins act predictably as we observed a relatively good correlation (*R*^2^ = 0.78 (CHO), 0.79 (HEK)) between MFE and RFP expression changes upon linear regression analysis (Figure 2F). Stable hairpins (low MFE) mediated a strong repression, whereas weak hairpins (high MFE) reduce less or even upregulate expression levels. In addition, fitting a curve by a non-linear least squares approach represents the observed relationship between MFE and protein expression levels better and thus potentially offers a tool to predict efficiencies of future RgEs (Supplementary Figure S3). No general correlation between RFP regulation and the GC-content of the RgEs could be observed (Supplementary Figure S4). Nevertheless, comparing elements of the same strength and position by subtraction of fold change observed with high GC element from the fold change of low GC equivalent element revealed that a high GC-content generally mediates a stronger downregulation of RFP expression than the equivalent RgE with low GC-content (Figure 2G) by an average of −0.12-fold in CHO and −0.17-fold in HEK cells. Interestingly, but for unknown reasons, RgE 12 and 13 as the only pair (similar MFE, same position but different GC content) in these experiments did not show any differences. We also found that elements closer to the 5′-cap are more potent in repressing protein translation as suggested (31), i.e. RgE 3 shows higher downregulation at position 5 than similarly strong elements at position 15, such as RgE 12 and 13.

Advanced RNA secondary structures, so-called ‘kissing hairpin’ or ‘KH’ (50), were generated to see whether we could push the regulation efficiencies even further down or completely repression expression. Therefore, KH sequences that harbor two RNA hairpin structures adjacent to one another plus complementary sequences in the hairpin loop were designed. Screening KH-mediated regulation efficiencies in CHO-K1 cells in the same set-up as the RgEs before demonstrated that the principle of tandem hairpins works as KH 1 showed a stronger regulation efficiency than the individual hairpins, RgE 7 and 16. Still, although the overall highest down-regulation was observed with KH 2, these elements did not further reduce RFP expression substantially stronger as the single hairpin RgEs (Supplementary Figure S5).

### RgEs impact translational rate, but also cause mRNA degradation

**Figure 3.**
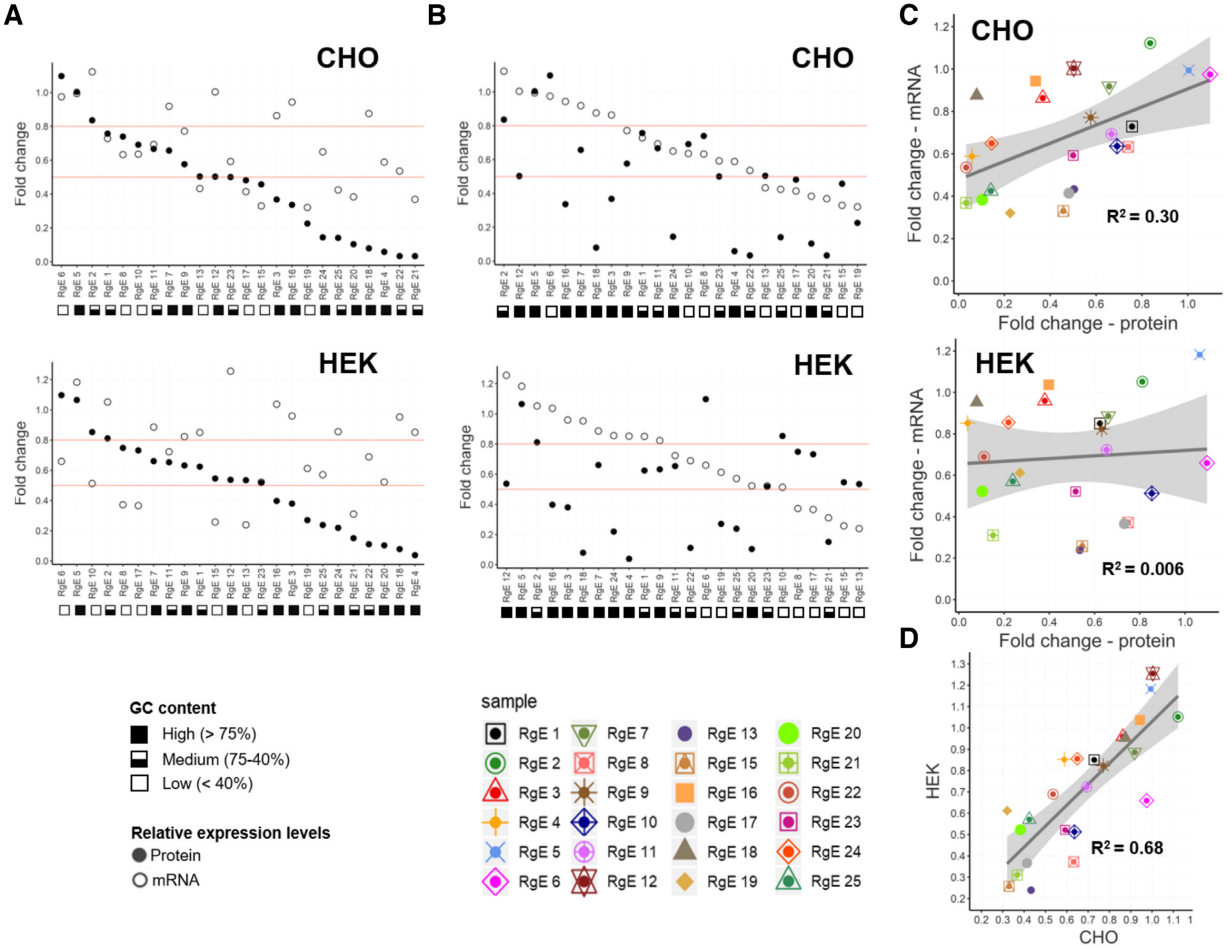
mRNA expression level analysis. (**A** and **B**) Observed average fold changes in RFP expression levels on the protein (taken from Figure 2C and D, full dots; *n* = 3 independent samples each) and mRNA level (open dots; *n* = 2 independent samples each; see Supplementary Figure S6 for individual values) for CHO (top) and HEK (bottom) cells ordered in decreasing order for protein expression levels (A) and mRNA levels (B). RNA to protein fold change ratio is depicted in Supplementary Figure S7. The red lines highlight specific fold changes that should simplify evaluation of fold change and impact of mRNA levels. (**C**) Average fold changes of mRNA levels plotted against fold changes on protein level for CHO (top) and HEK (bottom) cells. (**D**) Average mRNA fold changes of CHO versus HEK cells. Gray area shows standard error.

Messenger (m)RNA levels of RFP and BFP were determined to confirm that the RgEs impact the translational process. Therefore, qPCR on both CHO and HEK isolated RNA samples was performed. In fact, RFP mRNA levels were found to be different compared to the unregulated CMV control (Supplementary Figure S6), demonstrating that RFP mRNAs were selectively degraded due to the presence of RgEs. Interestingly, comparing the mRNAs changes for the two tested cell types showed that there is a fairly good correlation (*R*^2^ = 0.68) (Figure 3D). This indicates that the RgE-caused reduction of RFP mRNAs is not random, but results from one (or more) common mechanism(s) in mammalian cells. Nevertheless, when plotting the relative changes of mRNA levels to the observed changes in protein levels, no obvious relationship between these two factors could be detected (Figure 3A–C) as either no or only poor correlation was observed (*R*^2^ = 0.006 (HEK) or 0.30 (CHO)). These findings support the hypothesis that, while there is some impact on the mRNA stability, the RgEs act primarily by influencing the translation rate. Further substantiation was provided by calculating the RNA to protein fold change ratio which revealed that the protein expression levels and not the RNA levels are the major impacting factor (Supplementary Figure S7). Interestingly, there is an indication that a higher GC-content of the RgE had a protective effect on the mRNA levels, as six out of eight RgEs in CHO and nine out of eleven RgEs in HEK293, with an mRNA fold change above 0.8 (upper red line) had a GC-content >75%. In contrast, four out of seven RgEs in CHO and five out of one RgEs in HEK293 with a fold change below 0.5 (lower red line) had a GC-content <40% (Figure 3B). Lastly, we hypothesized that RNA hairpins are processed by the RNAi machinery as they mimic the natural target of RNase III DROSHA. However, testing several already evaluated siRNA sequences as integrated short hairpin (sh)RNA-RgE revealed no specific downregulation of the respective target mRNAs (Supplementary Figure S8).

### RgE-tuned heavy chain expression improves IgG yield and quality

**Figure 4.**
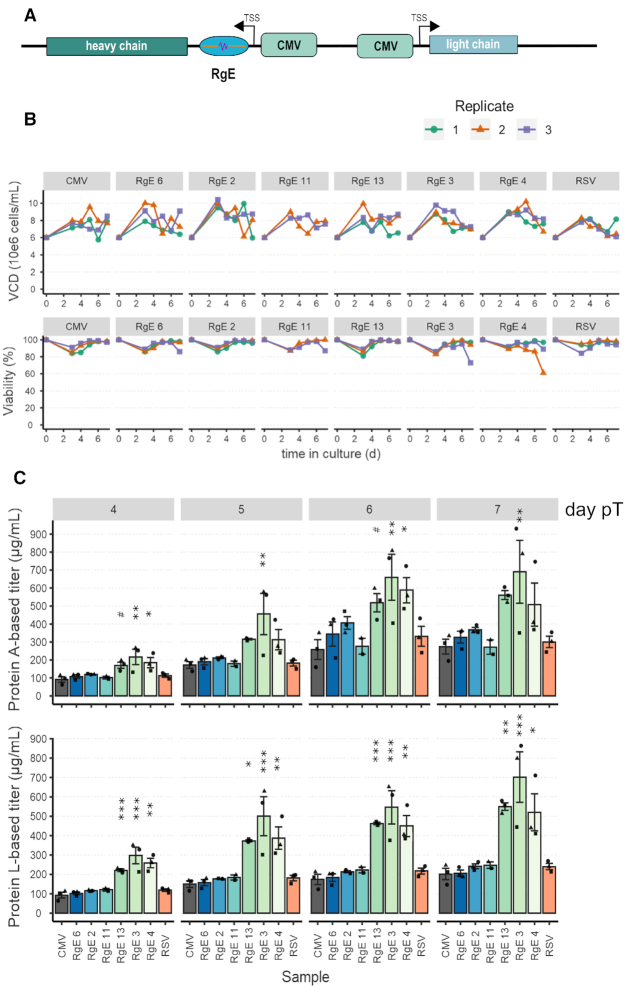
Using RgEs to tune HC expression levels to optimize recombinant production of an IgG. (**A**) Schematic overview of the expression construct. (**B**) Viable cell densities (VCD) and viabilities (%) of the transfected ExpiCHO™ cells (*n* = 3 independent samples each; RgE 11: *n* = 2). Columns represent different RgE constructs introduced. (**C**) Measured IgG titers on different days post transfection (pT) by either protein A- (top) or L-based (bottom) sensors. Samples are ordered by decreasing expression strength of the HC (*n* = 3 independent samples each; RgE 11: *n* = 2). Shapes of the data points indicate the respective replicates as depicted by the shapes in (B).

**Figure 5.**
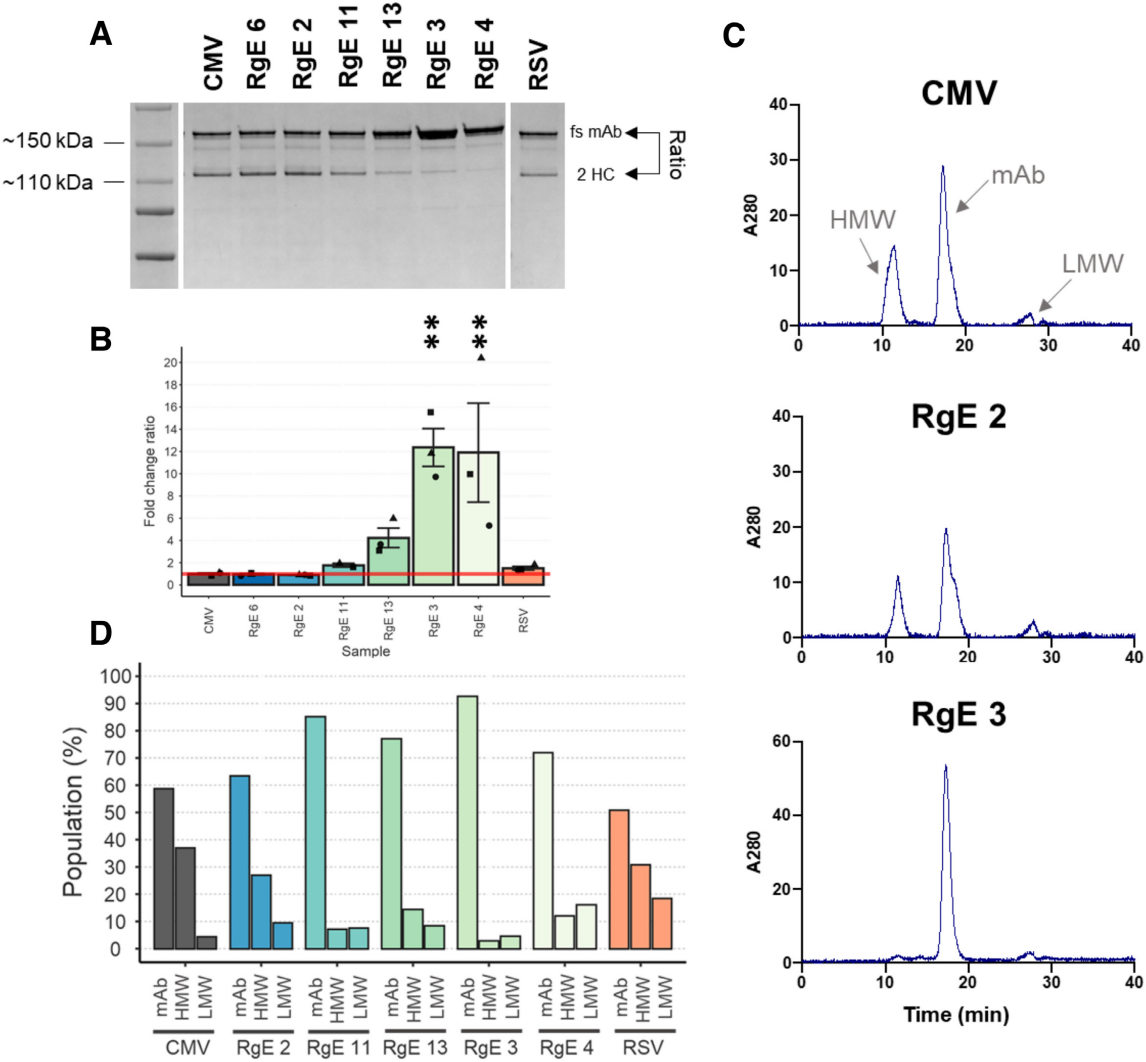
Analysis of product quality of the produced IgG. (**A**) Non-reducing sodium dodecyl sulphate-polyacrylamide gelelectrophoresis (SDS-PAGE) of protein-A beads purified IgG from the supernatant on day 6 post-transfection. fs = full size, 2 HC = heavy chain dimer. An image of the reduced plots can be found in Supplementary Figure S9b. (**B**) Calculated fold change (FC) of the ratio of fs to 2 HC from purified IgG from the supernatant on day 6 post-transfection referenced to sample CMV (*n* = 3 independent samples each; RgE 11: *n* = 2). Shapes of the data points indicate the respective replicates as indicated in Figure 4B. Gel pictures from all three replicates are shown in Supplementary Figure S9a. (**C**) Selected SEC chromatograms of protein A column purified IgGs produced. HMW = high molecular weight non-native fractions, LMW = low molecular weight non-native fractions, mAb = monoclonal antibody (wanted, native form of trastuzumab). All SEC chromatograms are shown in Supplementary Figure S10. (**D**) Calculated population distribution for respective RgE-produced IgGs based on peak areas shown as percentage of total peak areas for each chromatogram.

Employing RgEs to control the relative expression of the heavy chain (HC) of a trastuzumab monoclonal IgG1 (trade name Herceptin^®^) was used as a model system to assess the applicability of RgEs for improving titer and/or quality of the recombinantly produced antibody in CHO cells. The six RgEs 4, 3, 13, 11, 2 and 6, that mediate a down regulation to ∼5, 35, 50, 65, 85 and 110% of initial CMV expression rates (Figure 2), were cloned into the 5′-UTR of the HC gene (Figure 4A). Also, one control where the HC was expressed from an RSV promoter (slightly weaker promoter than CMV (51)) was included (‘RSV’). ExpiCHO™ cells were transiently transfected with these constructs and growth and IgG expression monitored (Figure 4B and C). No or only minor differences were seen in viable cell densities or viabilities. IgG titers were assessed with a Protein A and Protein L biosensor. Both sensors detect fully assembled IgGs, but bind to the different chains of the antibody. In both measurements, we found that the total titers were increased significantly in the samples where HC expression was reduced below 50%, with RgE 3 (∼35%) yielding the best expression results. RgE 3 samples showed a 2.5- (Protein A) – 3.5-fold (Protein L) increase of IgG in the supernatant at the end of the batch culture, corresponding to an increase of ∼400 or 500 μg/ml IgG concentration compared to the CMV sample. Moreover, analyzing purified IgG revealed that the ratio of full-size antibody to fragmented antibody was improved significantly by 12.4-fold with RgE 3, 11.9-fold with RgE 4 and 4.2-fold with RgE 13 (Figure 5A and B). Intracellular IgG fragments from day 6 post transfection were visualized by western blot, demonstrating that the HC expression decreases with the strength of the introduced RgEs. Moreover, increased signals for full-size and HC-LC fragments can be detected in the samples RgE 4 and 3, indicating a better assembly of the full-size antibody (Supplementary Figure S9). To further confirm these results of an improved qualitative product post purification, antibodies were expressed a second time, purified by Protein A column HPLC (to mimic industrial settings) and analyzed by size exclusion chromatography (SEC). The chromatograms (Figure 5 and Supplementary Figure S10) show a decrease of heavier and lighter non-native species, with the highest qualitative antibody product observed when tuning HC expression with RgE 3 resulting in 92.5% native full length antibody compared to 59% from the unregulated CMV (Figure 5C and D). Evaluation of the HC mRNA levels revealed no major differences of RgE-regulated expression samples compared to the CMV samples (Supplementary Figure S11), hinting again at a translational regulation mechanism. Most elements showed a comparable behavior as observed before (Figure 3), except for RgE 3 with slightly higher mRNA levels. The RSV samples had mRNA levels at slightly lower levels than those from the CMV promoter and also showed no significant differences to the CMV-mediated IgG secretion levels, suggesting that the HC expression was still too high to allow improved processing of IgGs. In fact, the RSV samples showed a very similar expression profile of both titer and product quality as their RgE equivalent RgE 2.

### Tuning of SUMF1 expression to achieve maximum activity of Arylsulfatase A

**Figure 6.**
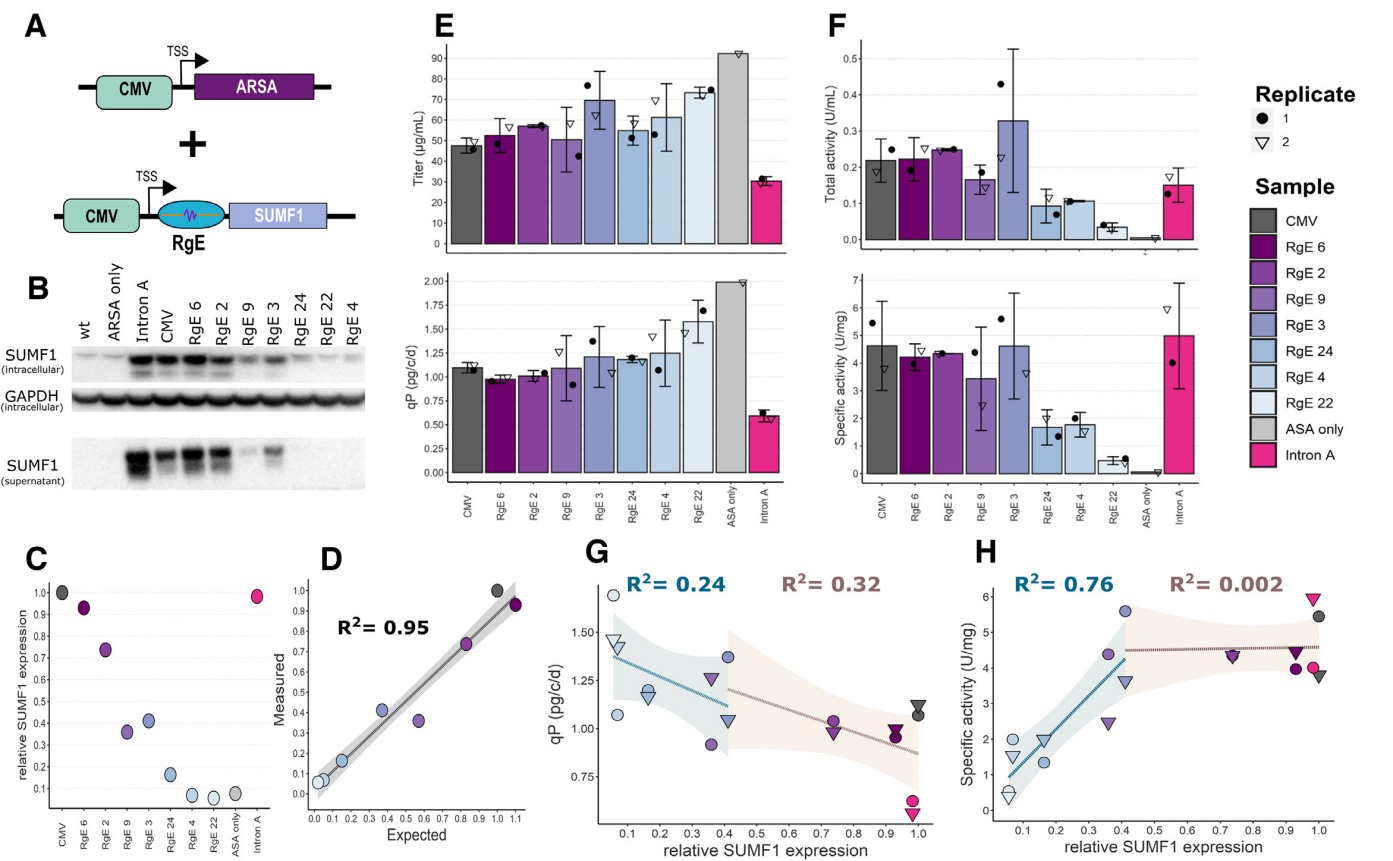
Tuning of helper protein SUMF1 levels impact ASA expression and activity. (**A**) Schematic overview of the expression strategy. (**B**) Western blot detection of SUMF1 levels intracellularly (from cell lysates) and secreted (supernatant). GAPDH was used as loading control. (**C**) Calculated relative SUMF1 expression levels (to CMV sample) based on intracellular and secreted SUMF1 signals form (B). (**D**) Measured and calculated SUMF1 levels based on the western blot versus the expected levels from Figure 2C. Gray area shows standard error. (**E**) Measured ASA titer (top) and calculated qP (bottom) values (*n* = 2 independent samples). Error bars show 95% confidence interval. (**F**) Measured ASA activity (top) and calculated protein specific activity (bottom) (*n* = 2 independent samples). Error bars as in (E). (**G**) ASA qP versus relative SUMF1 expression. *R*^2^ calculated by linear regression of low (blue) and high (brown) relative SUMF1 expressions. Colored areas show standard error. (**H**) ASA specific activity versus relative SUMF1 expression.

Co-expression of helper factors to improve productivity or product quality of mammalian cell factories is well established (52), but precise control and identification of the required expression levels is becoming more crucial in order to not waste cellular resources on unnecessarily high levels of such helper factors (10). Therefore, we sought to use RgEs to characterize and ideally optimize recombinant expression of ASA, a difficult-to-express therapeutic sulfatase, by tuning the expression of the helper factor SUMF1 (Figure 6A). Consequently, ASA was co-expressed (from the ARSA gene) with eight different translation levels of SUMF1 in ExpiCHO™ cells using RgEs 22, 4, 24, 3, 9, 2 and 6. An unregulated CMV sample, one sample including the CMV intron A in the 5′-UTR and one sample that only expressed ASA were used as controls. The RgE-tuned expression levels of SUMF1 were confirmed by western blot of the cell lysate and supernatant (SUMF1 is partially secreted (53)) 4 days post transfection (Figure 6B–D). Intriguingly, the expression levels matched well to the expected levels from previous experiments (Figure 2C), confirming the reliability of RgEs. After harvest on day 8 of the batch culture, ASA was purified. The results indicate that the titer of ASA decreased with increasing expression levels of SUMF1 from ∼80 to 37 μg/ml. These findings are substantiated when the ASA specific productivities (qP) were calculated showing that the highest qPs were achieved with ASA only expression (∼2 pg/c/d) or RgE 22 (∼1.6 pg/c/d) respectively, whereas lowest qPs were observed in the Intron A sample (∼0.6 pg/c/d) (Figure 6E). ASA activity was measured using a colorimetric assay and revealed that the total and protein specific ASA activity was low and almost undetectable when expressed alone, whereas it increased when co-expressed with SUMF1 to a maximum ∼ 4.5 U/mg or ∼0.2–0.3 U/mL ASA (Figure 6F). After achieving a level of SUMF1 expression of 0.4-fold relative to the CMV driven expression level, no further improvements in activity or yield were detected. Linear regression of relative SUMF1 levels versus qP or the specific activity revealed a negative trend with qP (*R*^2^ = 0.50), and a positive correlation with the specific activity (*R*^2^ = 0.63) (Supplementary Figure S12). Intriguingly, dividing the SUMF1 dosed samples in low (<0.4-fold) and high (>0.4-fold) relative SUMF1 expression demonstrated no specific differences on the qP as the negative trend persists (Figure 6G). Contrary, a clear difference was observed between the low and high group for the specific activity, showing a fairly good correlation (*R*^2^ = 0.76) between low SUMF1 expression levels and the activity, whereas no correlation (*R*^2^ < 0.01) could be observed above an expression level of 0.4-fold as mediated by RgE 3 (Figure 6H).

## DISCUSSION

In agreement with previous studies (27–28,31) we found that RNA secondary structures impact the translational rates from the respective mRNA in mammalian cells. The here generated RgEs were shown to linearly tune protein expression levels by approximately two orders of magnitude of the initial expression strength from an unregulated CMV promoter (Figure 2). From the three varied criteria we found that the MFE of the RgE was the most influencing regulation criterion (Figure 2F and Supplementary Figure S3). Thus, the toolbox can be employed in a predictable manner, i.e. introducing strong hairpins for a strong downregulation or weaker hairpins for a slight reduction or even upregulation. The biggest changes in protein expression levels happened between ∼−40 to −20 kcal/mol, in agreement with other publications (31,33). Further, our results show that elements with a high GC-content (>75%) generally mediate a slightly stronger repression than elements of the same thermodynamic stability with a low GC-content (<40%) (Figure 2G). These findings are supported by previous studies (31–33). Taken together, our results suggest to use MFE as a first parameter to roughly tune protein expression to a desired level and then diversify the GC-content and relative positions of the regulation element to achieve precisely attenuated expression levels.

Very similar regulation efficiencies of the RgEs were observed in two of the most used mammalian expression hosts for the production of therapeutic proteins, namely CHO and HEK293 cells (Figure 2). Eventually, these observations substantiate the rationale of intentionally interfering at the conserved translation process (54) to provide a toolbox that allows to reliably regulate protein expression levels in a variety of mammalian cell types. This idea is reinforced by other described techniques that regulate protein expression levels by translational control, such as different TIS (23) or uORFs (24). Moreover, these RgEs also represent an interesting module to engineer new or non-model mammalian/eukaryotic organisms that lack available molecular toolboxes (55).

Investigating more advanced structures (KH elements) in the 5′-UTR suggests that a stronger or even a complete repression of protein expression might not be possible with 5′-UTR RNA secondary structures (Supplementary Figure S5), in line with previous observations (31). Potentially the translational machinery can overcome such strong secondary structures, for example by ribosomal shunting (56,57). Still, there are many more RNA structures described that potentially could reduce expression levels further (26).

Evaluating the mRNA levels of the transgenes revealed that mRNAs with RgEs in the 5′-UTR are partially reduced but no obvious relationship of the differing mRNA levels and the observed protein expression levels could be identified (Figure 3). Also, no apparent correlation of the RgE-regulated HC mRNA levels (Supplementary Figure S11) and the HC protein expression levels (Supplementary Figure S9) could be detected. Hence these results suggest that the major action of regulating the protein expression levels is happening at the translational step. Noteworthy, we found an indication that mRNA levels of RgEs with high GC-content RNA hairpins are not affected as much as RgEs with a low GC content (Figure 3B). Endoribonuclease-mediated cleavage of UU or UA dinucleotides was described to regulate mRNA stability (58,59). As with decreasing GC-content in our RgEs many such dinucleotides are introduced, it seems plausible that the differences between low, medium and high GC elements are a result of such an endonuclease-mediated decay. Also different positions of the RgEs could affect mRNA stability. As the RgEs might also mimic target structures for the RNase III enzyme Drosha and Dicer, we tested whether RgEs can enter the RNAi pathway, but did not find any supporting hints for this (Supplementary Figure S8). Still, there are described examples of RNA hairpins that are cleaved by Drosha, but do not enter the RNAi pathway (60,61). While these considerations assume selective degradation of the mRNA strands, RNA secondary structures were also found in certain conformations to pause RNA polymerases (62,63) and thus potentially affect transcriptional rates. To identify the exact reason(s) for these varying mRNA levels, more detailed studies need to be carried out. Transfecting mRNA directly into the cells (64,65) would for example allow to omit the transcriptional step while still impacting translational rates and thus could shed light on the mechanisms that cause varying mRNA levels.

Tuning the expression levels of the recombinant product itself is especially important when the product is comprised of two or more subunits that require complex assembly processes, monoclonal IgG being a prominent example. Applying a panel of RgEs to tune down HC expression of trastuzumab showed that both the recombinant titer of secreted IgG as well as the proportion of full-size IgG in relation to aggregates and/or fragments could be improved significantly by a reduction of HC expression to ∼35% (Figures 4 and 5), hence supporting previously described examples (34–36). Especially, the increase in correctly assembled antibody and the accompanying decrease of heavier and smaller non-native molecular entities indicates that cells have problems with the assembly of full antibodies when overloaded with HC, thus reducing overall secretion on the one hand and resulting in secretion of incomplete molecules on the other hand (66). RgEs thus present themselves as a quick screening tool to determine the optimal relative expression rate of any multimeric protein of interest. We hypothesize that the here introduced RgE will also work reliably for generation of a stable production clone, as they are independent of any genomic environment or any other endogenous factors than the translation machinery. Intriguingly, we envision this toolbox of great value for the optimization of efficient expression of more difficult-to-express products, such as bispecific antibodies that heavily depend on the correct expression and assembly of multiple subunits (7).

Further interesting fields of application of this technology are metabolic and pathway engineering approaches where it has become more apparent that improved cellular phenotypes are likely achieved by precise control of protein/enzyme expression levels (10,67). RgEs could be used to control transgenic (over)expression of a cellular protein above the native level when upregulation is desired. Such a tuning strategy would be advantageous to find optimal expression levels and avoid metabolic overburdening or other detrimental effects on the cell, that might arise of expression from strong, unregulated promoters (i.e. CMV). In this regard, using i.e. the uORF strategy to control expression of a helper factor might be counterintuitive as the cells constantly express mini-peptides, and thus cellular resources, such as amino acids or energy (GTP/ATP), are occupied and missing elsewhere, i.e. for efficient production of the recombinant product. Co-expression of ASA with SUMF1 revealed that upon attainment of certain SUMF1 levels (∼40% of the initial, unregulated CMV expression) no substantial increase in protein specific or total activity could be gained. However, further increases in SUMF1 expression negatively impacted the specific ASA productivity. In this transient approach, the overall recombinant yield of active ASA was not significantly improved by RgE-tuned SUMF1 levels over ASA production with SUMF1 co-expression from an unregulated CMV promoter. Still, these findings substantiate the importance of co-factor tuning as we could detect maximum activities already at lower co-factor expression strengths (Figure 6). These results could provide future directions for an efficient production of this therapeutic sulfatase, in particular in view of generation of stable production cell lines. Here, the additional stress and the waste of resources for producing unnecessarily high levels of SUMF1 may well impact the likelihood of being able to isolate a stable high production clone with sufficient yield and quality for manufacturing. Evaluation of RgE-tuned SUMF1 levels and subsequent comparison to the expected values (Figure 6D) underlines the applicability of RgEs as reliable toolbox to tune and control transgene expression. Looking ahead and further afield, the use of the RgEs is not restricted to the fine-tuning of a single gene, but can easily be applied to multiple genes as predicted for instance by metabolic models to modulate pathways and enhance certain phenotypes. Considering the large number of recent studies and reviews that highlight the importance of gene dosage and balancing (i.e. 10–11,16,19,66–67,75) such tools will be of increasing importance in the future.

Enhanced designs of the RgE sequences, i.e. by including CRISPR/Cas9 target sequences in the DNA (68) or N^6^-methyladenosine sites in the RNA hairpin that can selectively be modified by novel CRISPR systems (69,70), could be employed to delete, diversify or destabilize the RgE structure. This potentially allows to rationally vary expression levels from one initial structure. Dedicated developments of these RgE structures could therefore add another control element that eventually can be employed in the construction of complex genetic networks or circuits (20,71).

Precise control of gene expression levels is not only important in mammalian cell factories, but is also an important success factor in gene therapy that is gaining ground on the pharmaceutical market (4). Contrary to monoclonal antibodies or other recombinant proteins where the administered dose can easily be controlled by adjustment of the protein concentration, tight control of gene expression from the viral vector in the target cells is often more difficult to achieve, but can be very crucial (72,73) to avoid severe side effects in the target tissue. We believe that the RgEs could efficiently be employed to tune the expression levels to a required expression state upon gene delivery. As the RgEs themselves do not depend on any endo- or exogenous factors other than the translation machinery, they should be applicable to any target cell types. Also, as they are small in size (∼90 bp maximum) they are compatible with viral vectors that are constrained in terms of the amount of genetic content (i.e. 4.7 kb in adenovirus-associated virus vectors).

In summary, we have presented the successful application of defined, synthetic RNA structures in the 5′-UTR of mRNAs to predictably tune protein expression levels within mammalian cells. Moreover, we showed that these RgEs can be employed to characterize and optimize recombinant production characteristics of CHO cells by intentionally regulating the expression of protein subunits or of a required helper factor. Together, these elements represent an easy-to-implement and reliable toolbox for future mammalian cell line engineering applications where precise control of protein expression levels is desired to improve the cellular phenotype, but they can also be employed in the investigation of basic biological processes.

## DATA AVAILABILITY

All sequences of the used RgEs are provided in the supplementary data. All other data is available from the corresponding authors upon reasonable request.

## Supplementary Material

gkaa847_Supplemental_File
