# Supplementary material for: Systematic use of synthetic 5′-UTR RNA structures to tune protein translation improves yield and quality of complex proteins in mammalian cell factories
Source: Nucleic Acids Res. 2020 Oct 13;48(20):e119. doi: 10.1093/nar/gkaa847 (PMC7672427; doi:10.1093/nar/gkaa847)
Supplement: gkaa847_Supplemental_File [file gkaa847_supplemental_file.pdf]

Supplementary table 1: Information on the RgE and Kissing Hairpins KH. Capital letters show complementary regions, small letters indicate the loop region. KH: red and blue letter colour indicates location of the first and second hairpin structure. Similarly, MFE of the KH is shown individual for both hairpins (separated by /), and the total in brackets.

Sample	MFE (kcal/mol)	Stem GC content (%)	Position	Sequence (5' --> 3')
RgE 1	-24.1	61	3	CAGATCCGCTAGCactcgagaGCTAGCGGATCTG
RgE 2	-15.9	66	7	TCCGCTAGCactcgagaGCTAGCGGA
RgE 3	-30.8	78	7	TCCGCTAGCGCGGCactcgagaGCCGCGCTAGCGGA
RgE 4	-63.8	75	7	TCCGCTAGCGCCTCCGGCGCGCACTCAactcgagaTGAGTGCGCGCCGGAGGCGCTAGCGGA
RgE 5	-10.4	100	15	CGGCCGactcgagaCGGCCG
RgE 6	-10.6	30	15	TTAGAACTAGactcgagaCTAGTTCTAA
RgE 7	-23.9	91	15	GCTGGCGCGCGactcgagaCGCGCGCCAGC
RgE 8	-25.1	33	15	TGTTCTACGTGACAATTAactcgagaTAATTGTCACGTAGAACA
RgE 9	-28.2	84	15	TCGGCCGTGCGCGactcgagaCGCGCACGGCCGA
RgE 10	-27.8	30	15	TCAAGTCTATATTGCTTTAGactcgagaCTAAAGCAATATAGACTTGA
RgE 11	-25.6	64	15	GTTCCCGTGCAACGactcgagaCGTTGCACGGGAAC
RgE 12	-29.7	92	15	GCGGCCGTGCGCGactcgagaCGCGCACGGCCGC
RgE 13	-29.6	29	15	TAGTCTATCTATTCATAACAGactcgagaCTGTTATGAATAGATAGACTA
RgE 14	-33.2	93	15	GAGCCGCCGCCGCGactcgagaCGCGGCGGCGGCTC
RgE 15	-32.5	32	15	TGATACTATTCCAATCCAATAGactcgagaCTATTGGATTGGAATAGTATCA
RgE 16	-34.8	87	15	GCGGCCGTGCGCGAGactcgagaCTCGCGCACGGCCGC
RgE 17	-34.1	36	15	TGCTATTCCAATCTTAACGAGAactcgagaTCTCGTTAAGATTGGAATAGCA
RgE 18	-62.5	84	15	GCCTCCGGCGCGCACTCACCGCCGCactcgagaGCGGCGGTGAGTGCGCGCCGGAGGC
RgE 19	-61.8	31	15	ACTAATCTAAGATCTTGCAATCTCACTAACACTCAATTTactcgagaAAATTGAGTGTTAGTGAGATTGCAAGATCTTAGATTAGT
RgE 20	-62.5	68	33	GCCTCCGGCGCGCACTCACCGCCGCactcgagaGCGGCGGTGAGTGCGCGCCGGAGGC
RgE 21	-35.4	71	3	CAGATCCGCTAGCGCGGactcgagaCCGCGCTAGCGGATCTG
RgE 22	-55.7	68	3	CAGATCCGCTAGCGCGGAGGCTACCactcgagaGGTAGCCTCCGCGCTAGCGGATCTG
RgE 23	-30.6	53	7	TCCGCTAGCTTCGACTTactcgagaAAGTCGAAGCTAGCGGA
RgE 24	-40.1	78	7	TCCGCTAGCGCGGCAGCCactcgagaGGCTGCCGCGCTAGCGGA
RgE 25	-39.2	52	7	TCCGCTAGCAGCGGCATTATAactcgagaTATAATGCCGCTGCTAGCGGA
KH 1	-23.9 / -34.8 (-59.13)	91 / 87	15	GCTGGCGCGCGactcgagaCGCGCGCCAGCCAACAACAAGCGGCCGTGCGCGAGagagctcaCTCGCGCACGGCCGC
KH 2	-23.9 / -62.5 (-87.4)	91 / 84	15	GCTGGCGCGCGactcgagaCGCGCGCCAGCCAACAACAAGCCTCCGGCGCGCACTCACCGCCGCagagctcaGCGGCGGTGAGTGCGCGCCGGAGGC
KH 3	-34.8/ -62.5 (-97.7)	87 / 84	15	GCGGCCGTGCGCGAGactcgagaCTCGCGCACGGCCGCCAACAACAAGCCTCCGGCGCGCACTCACCGCCGCagagctcaGCGGCGGTGAGTGCGCGCCGGAGGC
KH 4	-10.6 / -11.1 (-22.3)	30 / 30	15	TTAGAACTAGactcgagaCTAGTTCTAACAACAACAAATACTAGTGAagagctcaTCACTAGTAT

Supplementary table 2: Primer and oligo sequences used for cloning

Name	Sequence (5' --> 3')
TagRFP_fwd	CGAGAACATGTGTCATTAGTTCATAGCCCA
TagRFP_rev	GATACACATGTCAACACTCAACCCTATCTC
traz_fwd	AGACTGCGGCCGCGGTGGCGACCGG
traz_rev	GGACTGTTTAAACCTATGAACTAATGACCCCGTA
SUMF1_SacIX_fwd	TCGAGAATTCTTAATTAAAGCCTACACCGGGCGCGCCGA
SUMF1_SacIX_rev	AGCTTCGGCGCGCCCGGTGTAGGCTTTAATTAAGAATTC

Supplementary table 3: Primers used for quantitative real-time PCR. Primer efficiencies were determined from six dilution steps (1:10 dilutions) of the cDNA. NA = not applicable (for the transgenes).

Name	Sequence (5' --> 3')	Gene Symbol	Sequence accession number	Primer efficiency (%)	Amplification factor	Slope	y-intercept	R^2
BFP_qPCR_fwd	CACCATGAGCGAGCTGATTA	NA	NA	92.6	1.93	-3.51	13.29	0.983
BFP_qPCR_rev	TGCCGTAGAGGAAGCTAGT							
RFP_qPCR_fwd	CTAAGGGCGAAGAGCTGATTAAG	NA	NA	94.95	1.95	-3.45	14.32	0.997
RFP_qPCR_rev	TTCTGCTGCCGTACATGAAG							
HC_fwd	CAAGGACACCCTCATGATCTCCC	NA	NA	93.05	1.93	-3.50	11.22	0.991
HC_rev	GTGAGGACGCTGACCACAC							
gapdh_qPCR_fwd	AACTTTGGCATTGTGGAAGG	Gapdh	NM_001244854.2	91.35	1.91	-3.55	14.14	0.995
gapdh_qPCR_rev	ACACGTTGGGGGTAGGAACA							
DNMT1_fwd	TTGAATCCCCTCCCAAGACC	Dnmt1	NW_003613752.1	102.97	2.03	-3.25	20.29	0.978
DNMT1_rev	TCAAGTTGCTCCAGGACCTT							
DNMT3a_fwd	TGCCAGAACTGTAAGACTGC	Dnmt3a	NW_003613640.1	95.93	1.96	-3.42	18.63	0.988
DNMT3a_rev	GTAGCAGTTCCAGGGGTGTT							
CHD4_fwd	TTTGGCAGAAAGTCACCAGC	Chd4	NW_003613610.1	96.78	1.97	-3.40	17.03	0.993
CHD4_rev	AATGGTAGCTGGGAGTCGAG							
RAD21_fwd	CCAGGAGTCAGTGATGGAGG	Rad21	NW_003613668.1	101.76	2.02	-3.28	18.25	0.992
RAD21_rev	ATCTCCATCTGCTCGACCTG							
HPRT1	assay ID: Hs.PT.58v.45621572 (IDT Inc.)	HPRT1	NM_000194(1)	95.66	1.96	-3.43	16.34	0.976

Supplementary table 4: Antibodies used for western blots. AB = antibody

For visualization of	Used as	Dilution	Antibody	Conjugate	Catalouge Nr.	Provider
trastuzumab	primary AB	1:1000	mouse anti-human IgG (H+L)	/	#SAB3701329	Sigma-Aldrich, USA
trastuzumab	secondary AB	1:10000	goat anti-mouse IgG (H+L)	Alexa Fluor® 680	#A-21057	Thermo Fisher Scientific, USA
SUMF1	primary AB	1:1000	goat anti-human SUMF1 IgG	/	#PA5-19195	Thermo Fisher Scientific, USA
SUMF1	secondary AB	1:1500	rabbit anti-goat IgG (H+L)	Horseradish peroxidase	#A27014	Thermo Fisher Scientific, USA

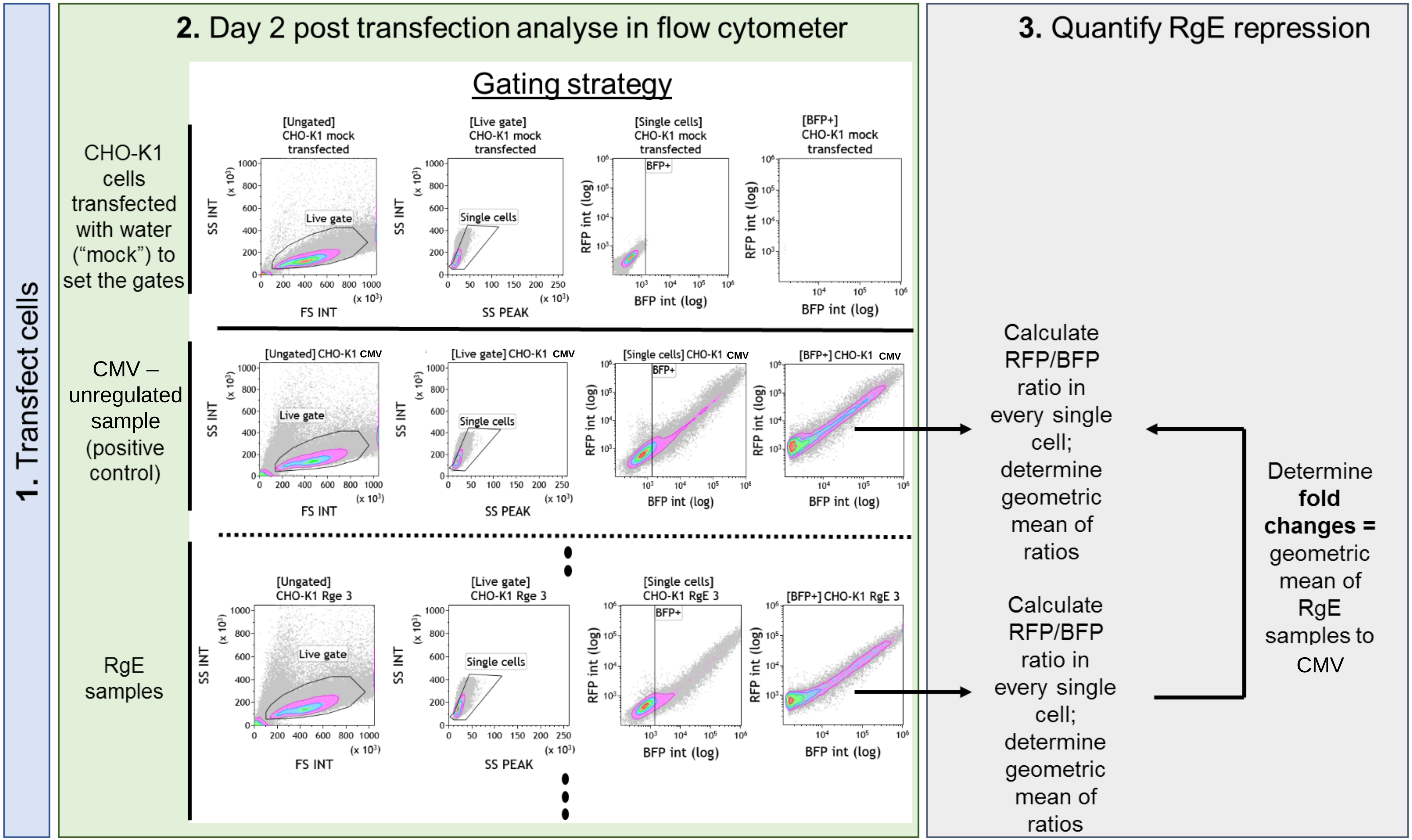

Supplementary figure 1: Work flow and gating strategy to determine RgE repression efficiency.

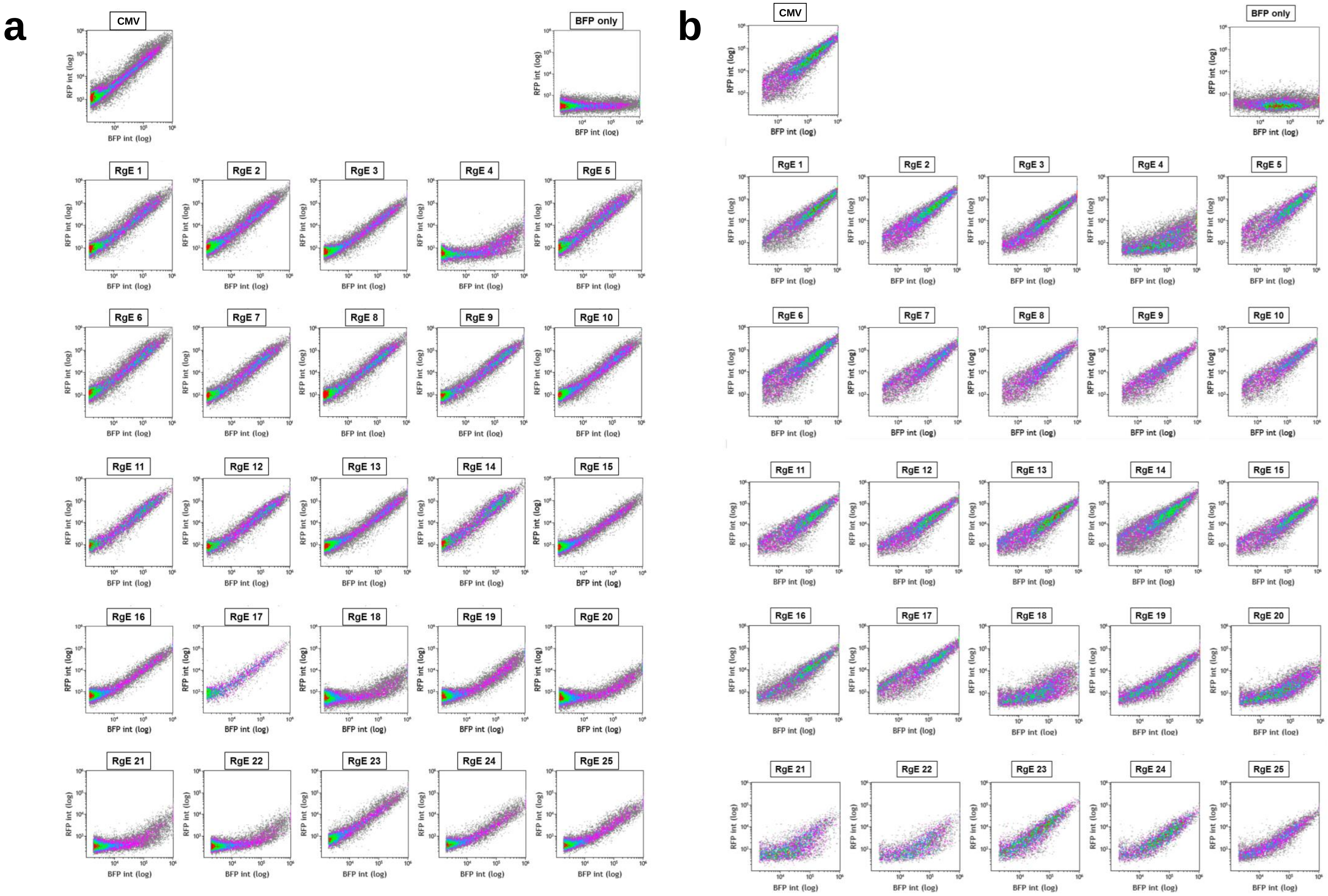

Supplementary figure 2: Flow cytometry charts of transfected (a) CHO cells and (b) HEK cells. Each plot depicts one replicate. Other replicates are not shown to reduce space requirements.

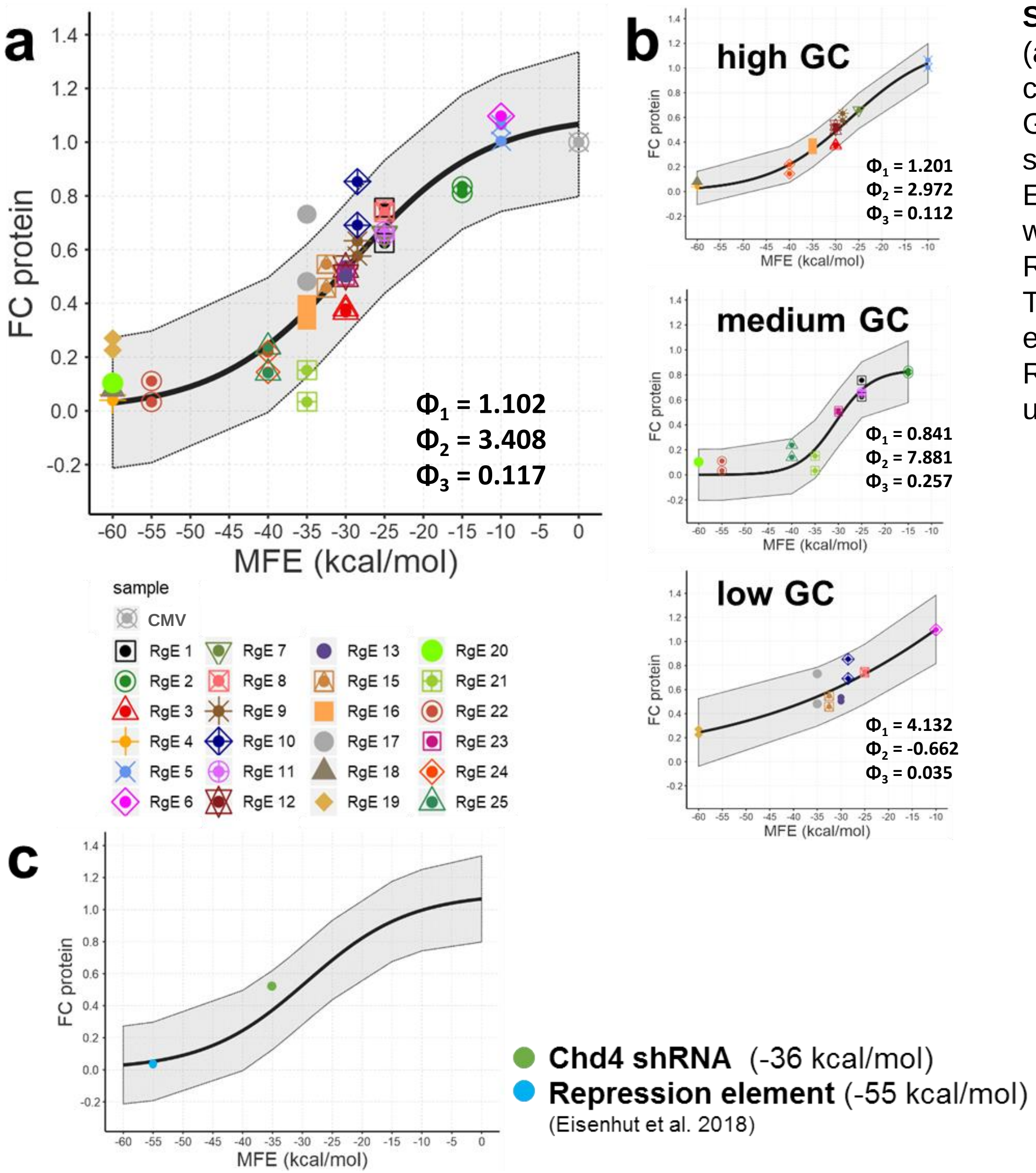

Supplementary figure 3: Predictable regulation by the RgEs based on MFE. (a) Non-linear least square (nls) modeling* (black line) of all RgEs. All calculated fold changes (from CHO and HEK) were used for the modeling. Grey area shows the prediction interval. (b) Modelled curves of RgEs separated by GC content. High > 75%; medium 75-40%; low < 40%. (c) Evaluation of the predicted curve and interval of two independent RgE that were not included in the model calculation. Green point is the fold changes of RFP expression observed from the Chd4 siRNA hairpin (see supp. figure 7). The blue point is the regulation efficiencies of an RNA hairpin repressor element described in one of our previous publications. Briefly, a -55 kcal/mol RNA hairpin was cloned to repress BFP expression and RFP expression was used as reference.

* nls predictions based on the formula:

$$y = \frac{\Phi_1}{1 + e^{-(\Phi_2 + (\Phi_3 * x)}}$$

Eisenhut,P., Klanert,G., Weinguny,M., Baier,L., Jadhav,V., Ivansson,D. and Borth,N. (2018) A CRISPR/Cas9 based engineering strategy for overexpression of multiple genes in Chinese hamster ovary cells. Metabolic Engineering, 48, 72–81.

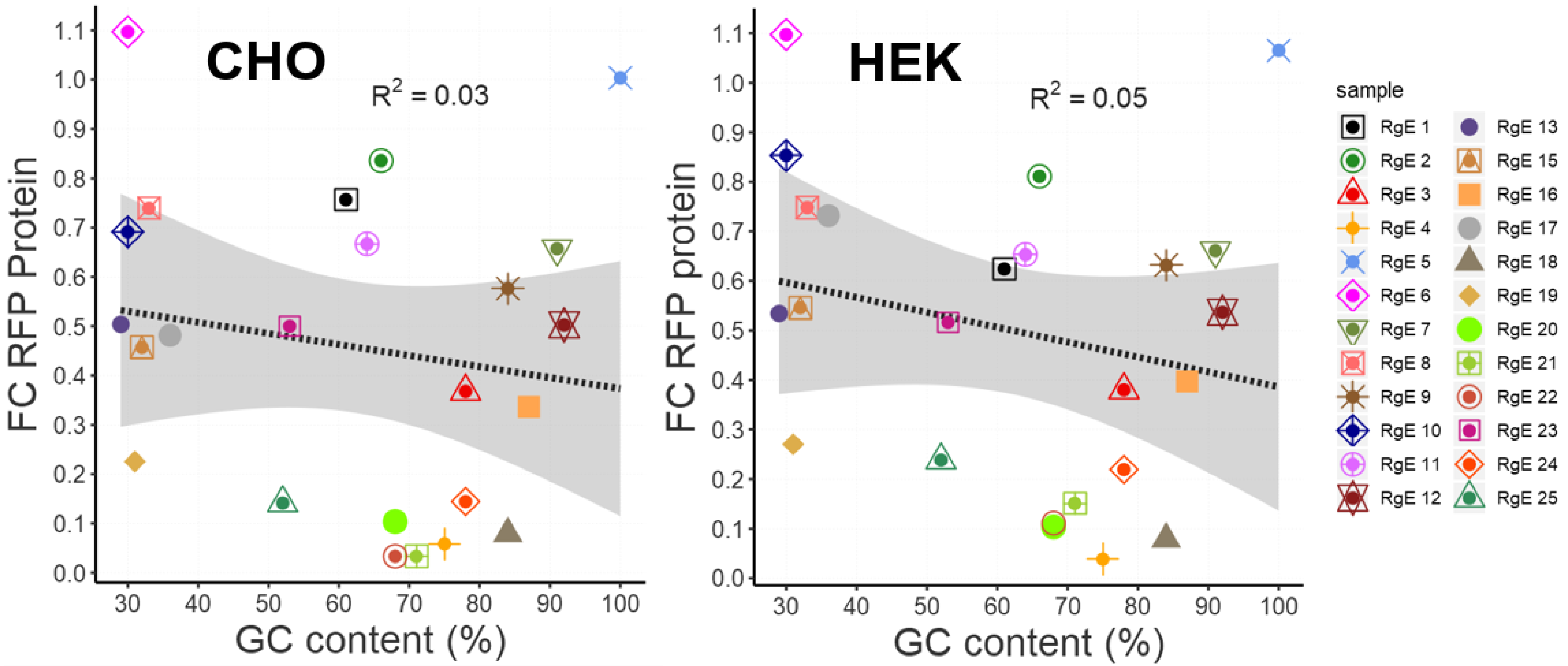

Supplementary figure 4: Average protein expression fold changes (FC) of CHO (left) and HEK (right) cell plotted against the GC-content of the respective elements. Grey area as in shows standard error of mean (SEM).

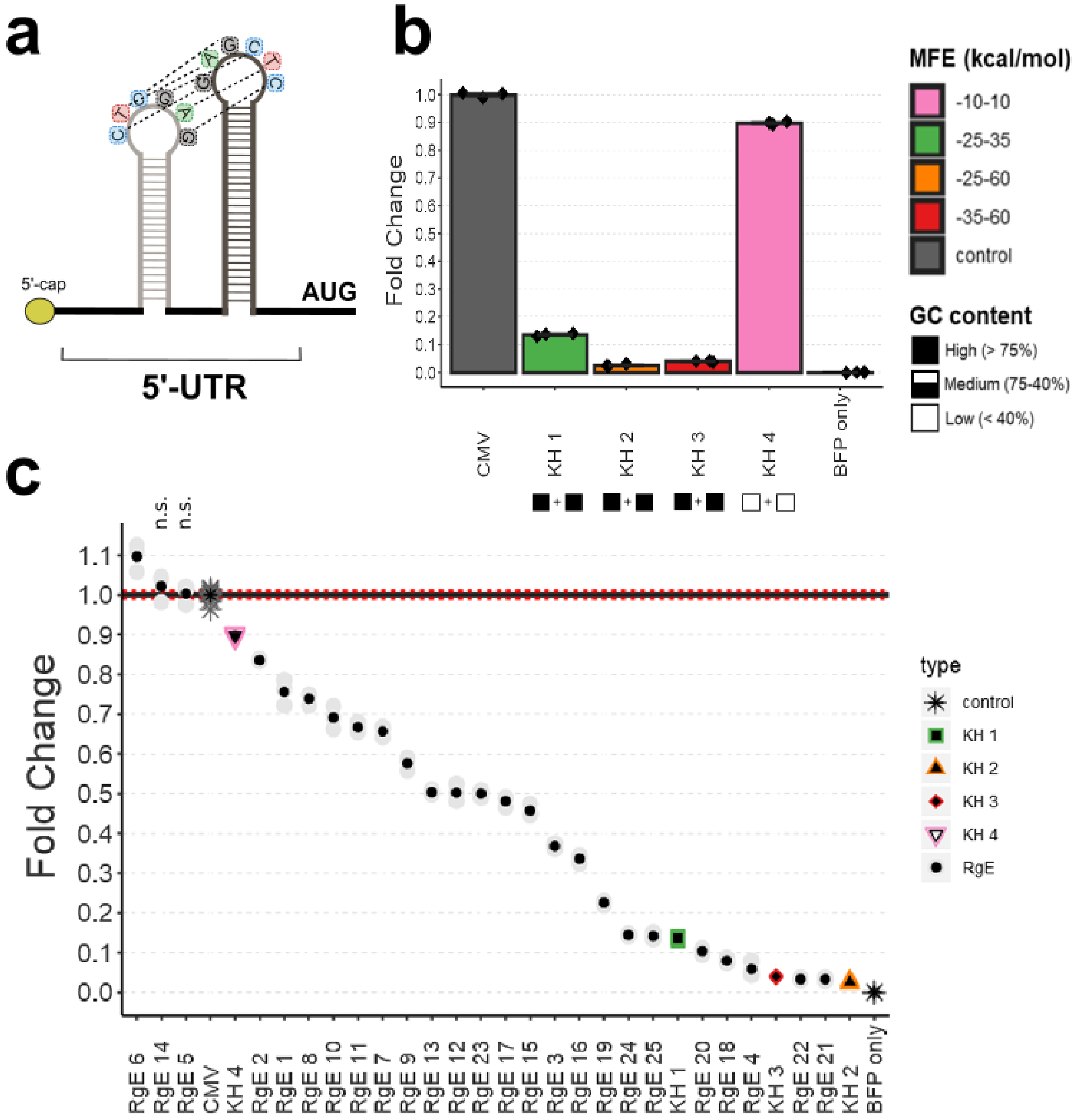

Supplementary figure 5: Kissing hairpin (KH) regulation elements. (a) Schematic representation of KH. Two hairpins are introduced into the 5'-UTR with complementary loop sequences. (b) Calculated fold changes of RFP/BFP expression ratio mediated by the KH structures in CHO cells. Bars show average, points show individual values of samples (n=3 independent samples each). GC-content of the individual hairpins indicated by the two boxes below each sample. (c) Comparing KH elements (in color) to RgE (grey) from Figure 2b. Statistical test in comparison to CMV sample.

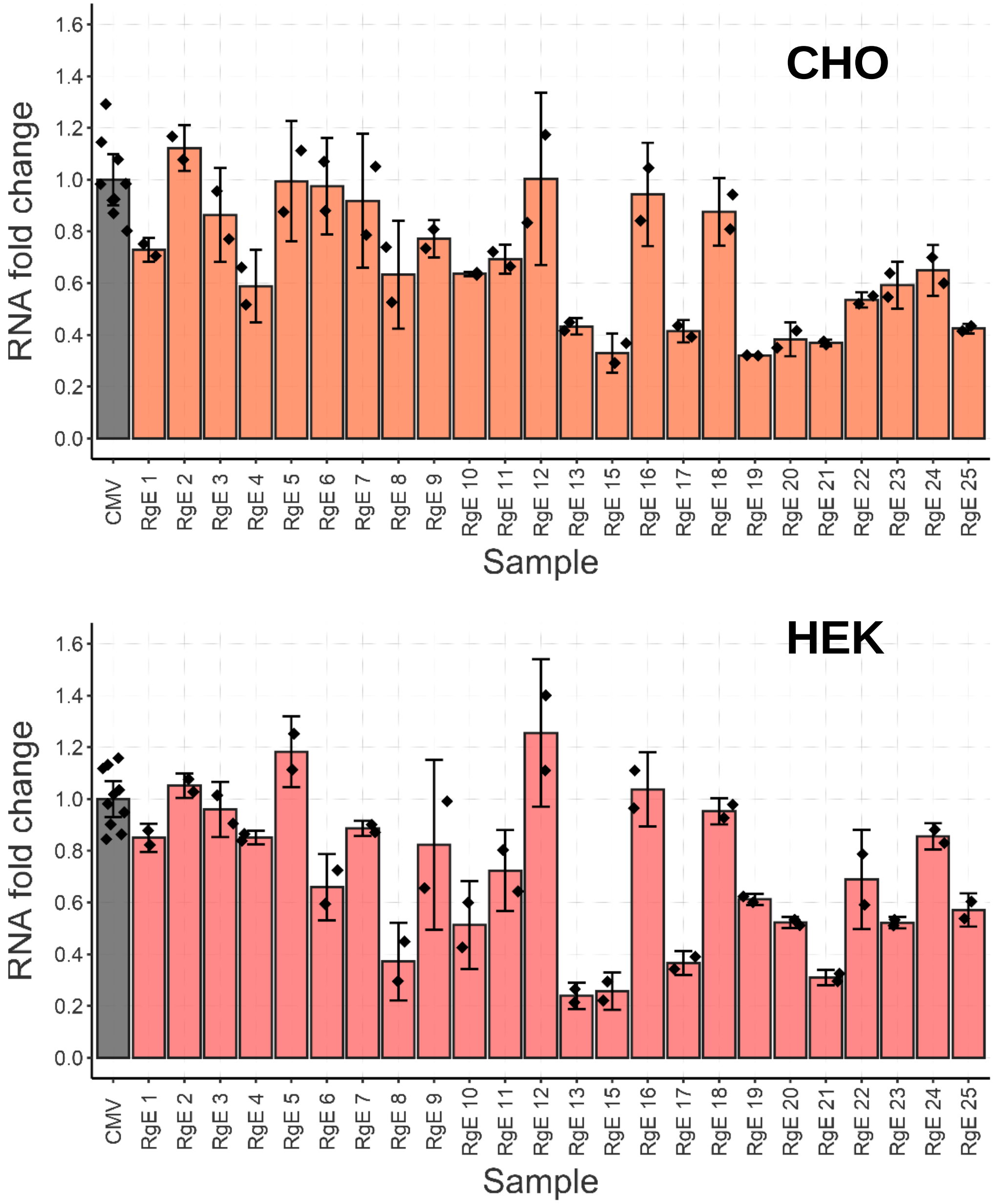

Supplementary figure 6: Average RNA fold changes for CHO (top) and HEK (bottom) cells. CMV sample is shown in grey. Error bars show the 0.95 confidence interval and dots the actual values of two replicates.

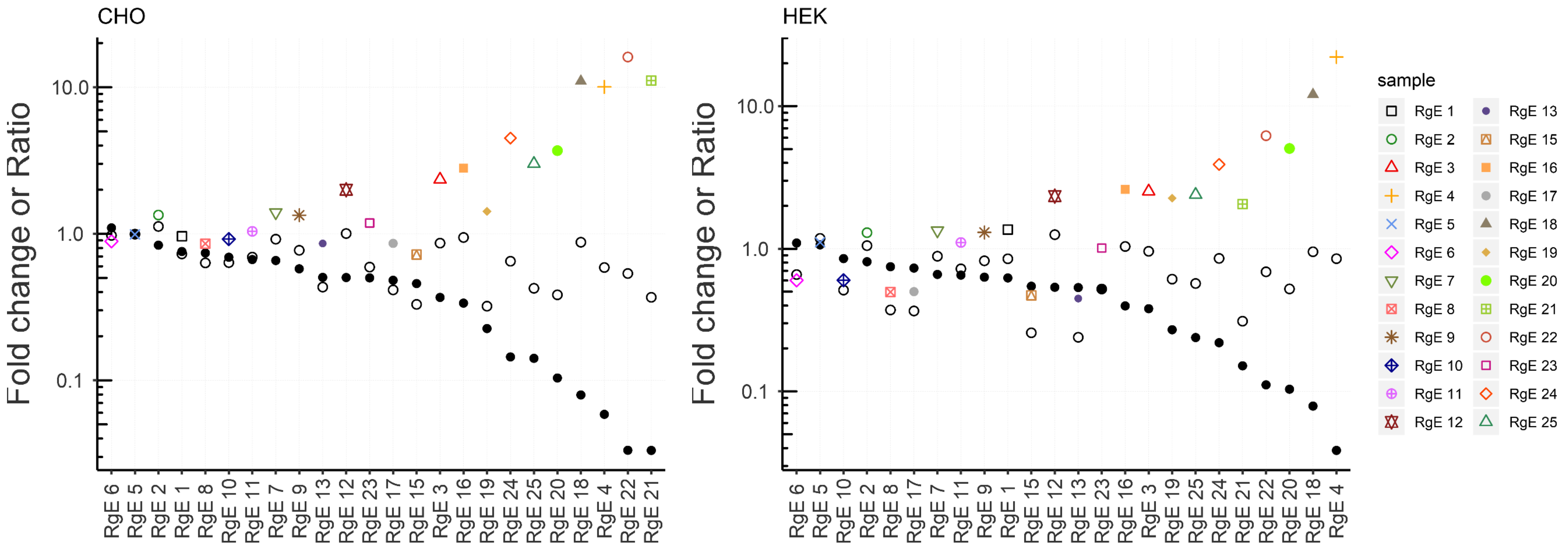

Supplementary figure 7: Observed average changes in RFP expression levels on the protein (taken from Figure 2c and d, full black circles; n=3 independent samples each) and mRNA level (open black circles; n=2 independent samples each; see Supp. Fig. 6 for individual values) for CHO (left) and HEK (right) cells ordered in decreasing order for protein expression levels. Colored shapes depict the ratio of RNA/protein fold changes for each individual sample.

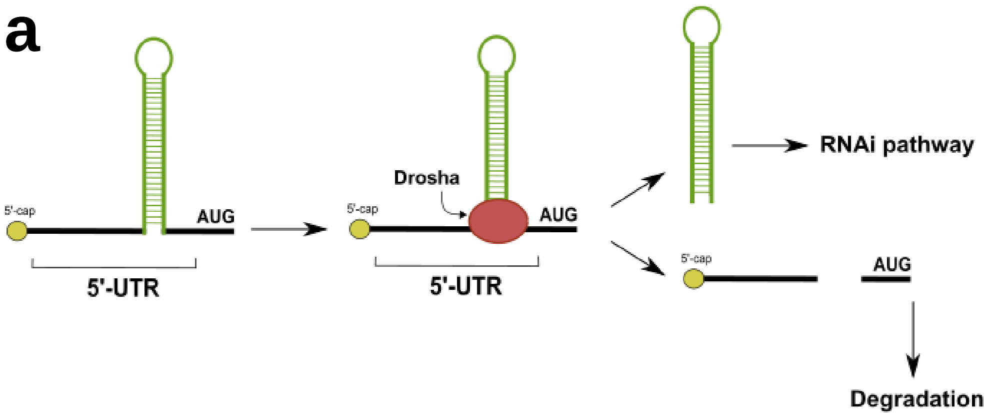

b

target	Sequence of the RgE (5' --> 3')
Rad21	***TATATAGTCACATAATTTC*** actcgagaGAAATTATGTGACTATATA
Chd4	***TGAGCTTTGGAGTCTCTGC*** actcgagaGCAGAGACTCCAAAGCTCA
DNMT1	***CTCACGCCATCTATGAAGA*** actcgagaTCTTCATAGATGGCGTGAG
DNMT3a	***GGACATTTGTATCTCATGT*** actcgagaACATGAGATACAAATGTCC

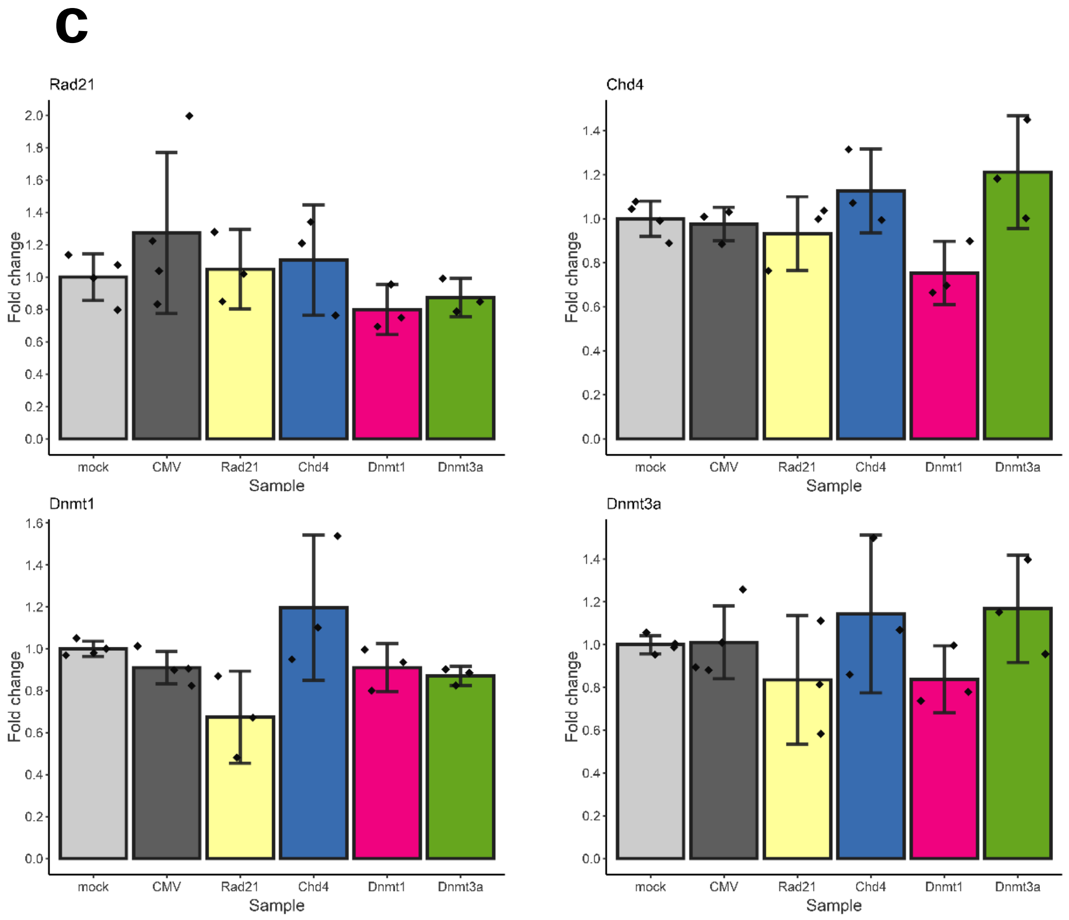

Supplementary figure 8: Testing whether RgEs are processed as short hairpin RNAs and enter RNAi pathway. (a) Schematic overview of the potential mechanism how RgEs could enter the RNAi pathway. (b) Four siRNA RgEs were tested. RgE sequences with siRNA sequences highlighted in bold and italic. These siRNAs were previously tested for their functionality (data not shown). (c) qPCR results. Graph title shows for which target qPCRs were performed (meaning which qPCRs primers were used); Samples indicate which RgEs were transfected into CHO cells. Relative fold changes calculated to the mock samples (mock = only water transfected). Error bars show 95% confidence interval.

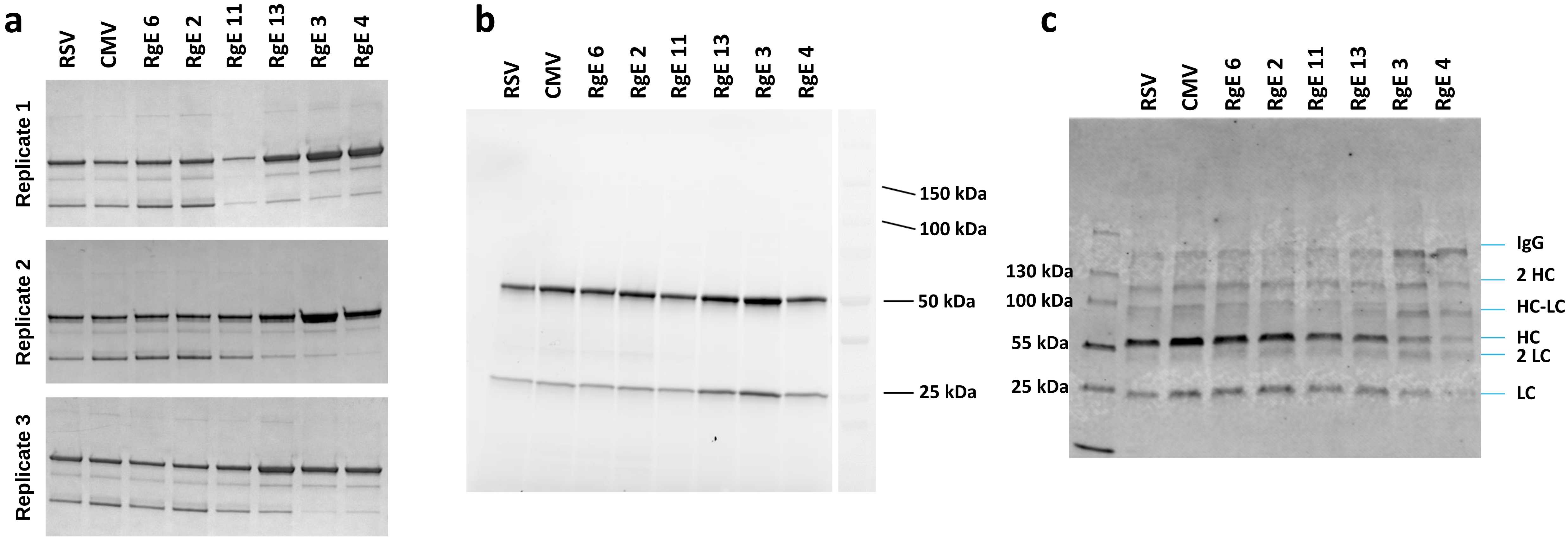

Supplementary figure 9: (a) Non-reducing SDS-Page of protein-A beads purified IgG from the supernatant on day 6 post transfection. (b) SDS-page of reduced protein-A beads purified IgG samples from day 6 post transfection (replicate 2). Ladder = Bio-Rad Precision Plus Protein™ All Blue Prestained Protein Standards. (c) Western blot detection of intracellular IgG fragments from samples from day 6 post transfection. Ladder = Thermo Scientific™ PageRuler™ Plus prestained protein ladder.

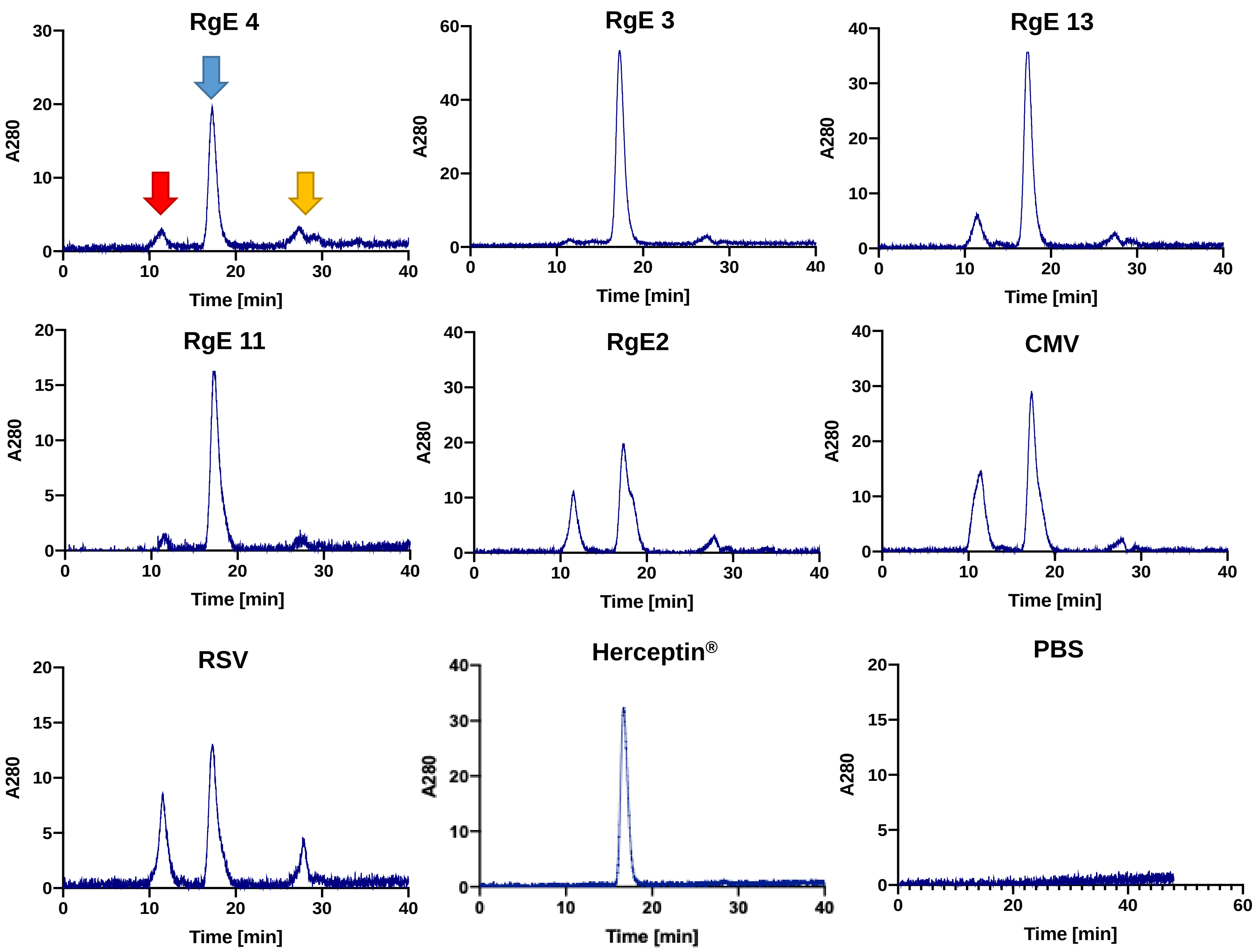

Supplementary figure 10:
Size-Exclusion Chromatography analysis of purified IgGs produced via modulation of HC expression levels with RgEs. Red arrow refers to heavy molecular weight non-native populations, blue arrow the wanted native full-length IgG and yellow arrow marks light molecular weight non-native populations. Herceptin® shows chromatogram of commercially available trastuzumab and PBS shows the background signal.

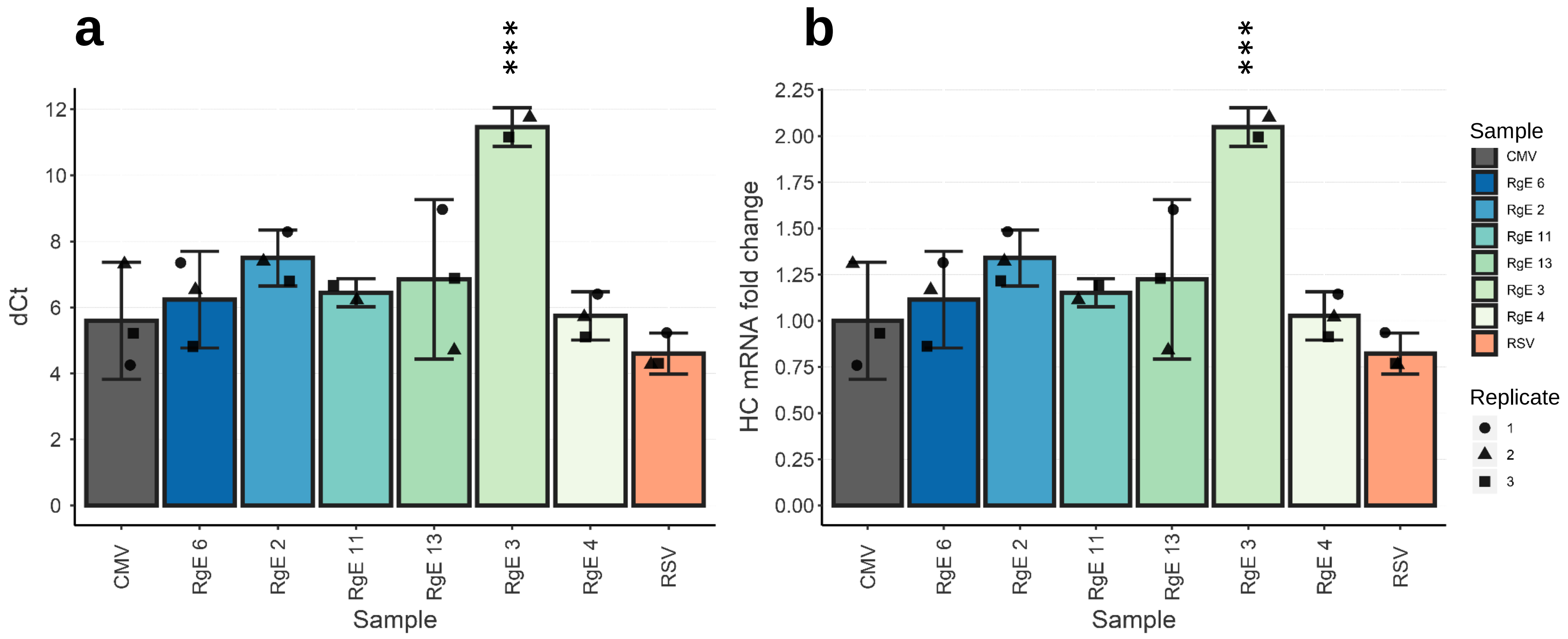

Supplementary figure 11: Heavy chain mRNA quantifications. (a) Calculated dCt from isolated RNA at the end of expression batch (n = 3; except RgE 11 and 3 where replicate was excluded due to faulty RNA isolation). Bars show calculated averages. Error bars show 95% confidence interval. Statistical differences compared to CMV sample. (b) Calculated fold changes in relation to CMV average. Bars, Error bars and statistics as in (a). Primer efficiencies and amplification factors are given Supp. table 3.

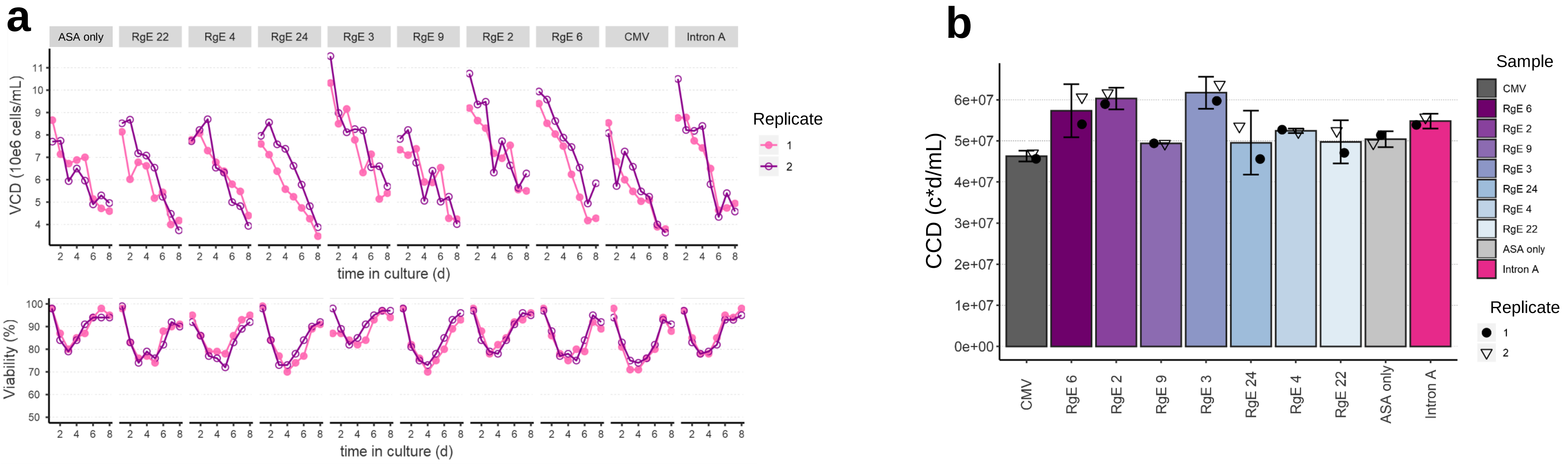

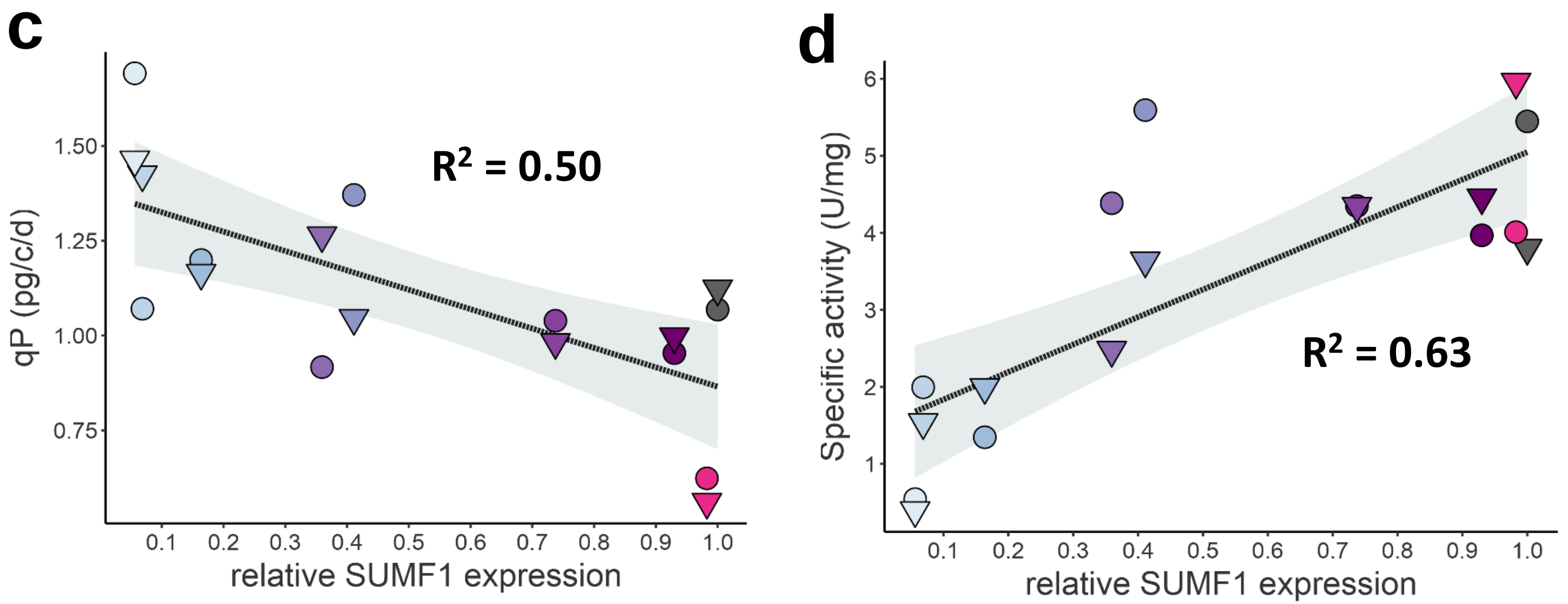

Supplementary figure 12: SUMF1 dosing and ASA expression.
(a) Viable cell densities (VCD) and viability (%) of transfected ExpiCHO™ cell lines followed over the ASA production process.
(b) Calculated cumulative cell days (CCD; area under the VCD curve) over the production process.(c,d) qP or specific activity versus relative SUMF1 expression levels. R^2 calculated by linear regression.
